# NLRX1 inhibits the early stages of CNS inflammation and prevents the onset of spontaneous autoimmunity

**DOI:** 10.1371/journal.pbio.3000451

**Published:** 2019-09-16

**Authors:** Marjan Gharagozloo, Shaimaa Mahmoud, Camille Simard, Kenzo Yamamoto, Diwakar Bobbala, Subburaj Ilangumaran, Matthew D. Smith, Albert Lamontagne, Samir Jarjoura, Jean-Bernard Denault, Véronique Blais, Louis Gendron, Carles Vilariño-Güell, A. Dessa Sadovnick, Jenny P. Ting, Peter A. Calabresi, Abdelaziz Amrani, Denis Gris

**Affiliations:** 1 Department of Pharmacology and Physiology, Faculty of Medicine, Université de Sherbrooke, Sherbrooke, Quebec, Canada; 2 Department of Neurology, Johns Hopkins University, Baltimore, Maryland, United States of America; 3 Department of Chemical Engineering and Biotechnological Engineering, Université de Sherbrooke, Sherbrooke, Quebec, Canada; 4 Department of Anatomy and Cell Biology, Faculty of Medicine, Université de Sherbrooke, Sherbrooke, Quebec, Canada; 5 Department of Neurology, Faculty of Medicine, MS Clinic, Université de Sherbrooke, Sherbrooke, Quebec, Canada; 6 Department of Medical Genetics, University of British Columbia, Vancouver, Canada; 7 Department of Microbiology and Immunology, Lineberger Cancer Center, University of North Carolina at Chapel Hill, Chapel Hill, North Carolina, United States of America; 8 Department of Neuroscience, Johns Hopkins University, Baltimore, Maryland, United States of America; 9 Department of Pediatrics, Faculty of Medicine, Université de Sherbrooke, Sherbrooke, Quebec, Canada; UCSD, UNITED STATES

## Abstract

Nucleotide-binding, leucine-rich repeat containing X1 (NLRX1) is a mitochondria-located innate immune sensor that inhibits major pro-inflammatory pathways such as type I interferon and nuclear factor-κB signaling. We generated a novel, spontaneous, and rapidly progressing mouse model of multiple sclerosis (MS) by crossing myelin-specific T-cell receptor (TCR) transgenic mice with *Nlrx1*^*−/−*^ mice. About half of the resulting progeny developed spontaneous experimental autoimmune encephalomyelitis (spEAE), which was associated with severe demyelination and inflammation in the central nervous system (CNS). Using lymphocyte-deficient mice and a series of adoptive transfer experiments, we demonstrate that genetic susceptibility to EAE lies within the innate immune compartment. We show that NLRX1 inhibits the subclinical stages of microglial activation and prevents the generation of neurotoxic astrocytes that induce neuronal and oligodendrocyte death in vitro. Moreover, we discovered several mutations within *NLRX1* that run in MS-affected families. In summary, our findings highlight the importance of NLRX1 in controlling the early stages of CNS inflammation and preventing the onset of spontaneous autoimmunity.

## Abstract 2

NLRX1 is a guardian protein that inhibits the inflammatory response of glial cells within the central nervous system and prevents the onset of a spontaneous multiple sclerosis–like disease in mice. This study uses a novel mouse model to provide mechanistic insights into the neurodegenerative origin of multiple sclerosis.

## Introduction

Multiple sclerosis (MS) is a neurological disease that affects young adults, leading to long-term disabilities with a staggering societal cost [1]. It is associated with inflammation-driven demyelination and neurodegeneration within the central nervous system (CNS) [1,2]. Over the last century, multiple studies have confirmed the autoimmune nature of MS [3]. Infiltration of various subsets of myelin-specific T and B cells precedes the development of inflammatory foci, demyelinating plaques, and axonal damage [2]. Despite the extensive efforts to define MS immunopathology, the origin of the disease is still a matter of debate.

Two main models have been proposed to explain the etiology of MS: outside-in and inside-out models. According to the first model, MS is primarily caused by aberrant peripheral immune responses outside of the CNS in the secondary lymphoid organs such as spleen and lymph nodes. The overactivation and infiltration of the autoreactive T and B cells into the CNS cause inflammation and progressive demyelination [4]. This model gave rise to the many of the contemporary disease-modifying therapies [5]. The inside-out model presents the idea that MS is primarily initiated by neurodegenerative processes in which oligodendrocyte and/or neuronal injury or death triggers the CNS inflammation in the absence of a direct immune attack [4,6,7]. This inflammation leads to the drainage of CNS antigens into secondary lymphoid organs and consequent activation of autoreactive T and B cells [6].

In both models, inflammation is present at all stages of the disease. It is triggered either by the infiltration of peripheral immune cells into the CNS or by the activation of CNS-resident cells, including microglia and astrocytes. Both innate and adaptive immune responses are involved in potentiating demyelinating neuroinflammatory disease in MS [1].

In our research, we looked into the mechanism of activation of microglia and astrocytes in the CNS. These cells can participate in the first line of the immune response by recognizing pathogens and/or danger signals via pattern-recognition receptors (PRRs), such as the nucleotide-binding oligomerization domain, leucine-rich repeat containing proteins (NLRs) [8].

NLRs regulate both innate and adaptive immune responses [4]. In the context of neuroinflammation, some NLRs such as the nucleotide-binding oligomerization domain, leucine rich repeat and pyrin domain containing 1 (NLRP1) and NLRP3 promote the development of experimental autoimmune encephalomyelitis (EAE), a mouse model of MS [9,10], whereas anti-inflammatory NLRs such as nucleotide-binding, leucine-rich repeat containing X1 (NLRX1) and NLRP12 inhibit CNS inflammation [11,12]. NLRX1 is a recently characterized member of the NLR family that is ubiquitously expressed and uniquely localized to the mitochondria. Since its discovery, NLRX1 has been implicated in multiple pathophysiological processes, including oxidative damage, mitochondrial dynamics, and cell death [13–17]. NLRX1 also inhibits major pro-inflammatory pathways such as type I interferon [15,18,19] and toll-like receptor (TLR)-mediated nuclear factor κB (NF-κB) signaling [20,21]. Eitas and colleagues observed more severe EAE and enhanced tissue damage in *Nlrx1*^*−/−*^ mice compared with wild-type (WT) [11]. They reported that microglia from *Nlrx1*^*−/−*^ mice released more pro-inflammatory cytokines and chemokines [11], suggesting the protective role of NLRX1 in the progression of MS.

Several T-cell receptor (TCR) transgenic mouse models have been developed to study the pathogenic role of T cells in MS [22]. Surprisingly, high numbers of myelin-specific T cells are not sufficient to induce spontaneous EAE (spEAE) in 2D2 mice [23], suggesting that other mechanisms are necessary to break immunological tolerance and induce CNS autoimmunity.

In the current study, we demonstrate that in the absence of NLRX1, the 2D2 mice develop a severe spEAE. We provide evidence of subclinical CNS inflammation in asymptomatic *Nlrx1*^*−/−*^ 2D2 mice that contributes to the generation of neurotoxic glia and the death of neurons and oligodendrocytes.

## Results

### *Nlrx1*^*−/−*^ 2D2 mice develop spEAE, which is associated with CNS inflammation and demyelination

**Fig 1:**
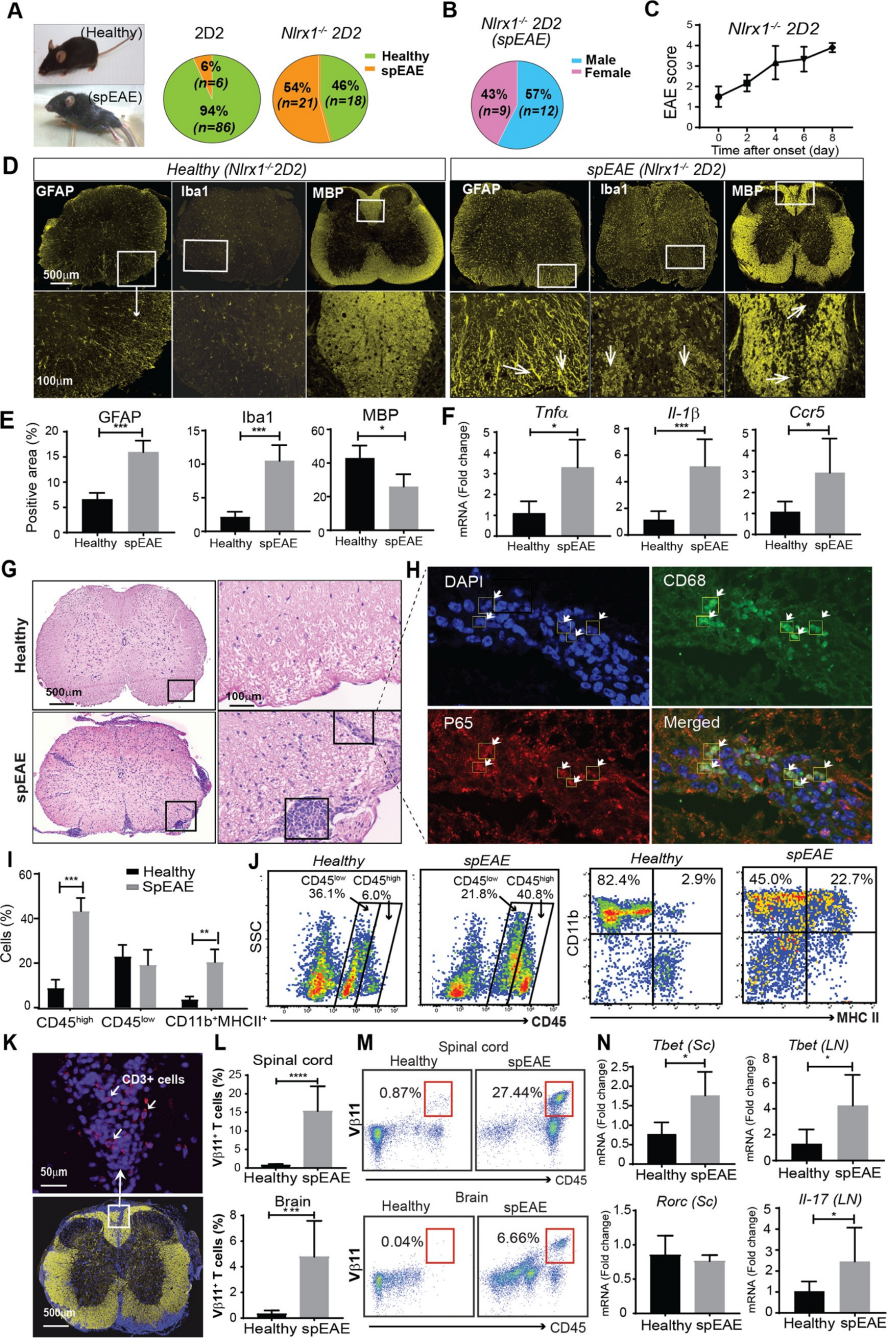
*Nlrx1*^*−/−*^ 2D2 mice develop spEAE, which is associated with CNS inflammation and demyelination. **(A)** The frequency of spEAE in 2D2 and *Nlrx1*^*−/−*^ 2D2 mice. (**B)** The frequency of spEAE in male and female *Nlrx1*^*−/−*^2D2 mice. (**C)** Clinical score of *Nlrx1*^*−/−*^ 2D2 mice, showing a progressive EAE from the onset (*n* = 6). (**D)** A representative immunofluorescent staining of spinal cords from healthy *Nlrx1*^*−/−*^2D2 and spEAE *Nlrx1*^*−/−*^2D2 mice for GFAP, Iba1, and MBP markers. White arrows show astrogliosis (GFAP), microgliosis (Iba1), and focal demyelinating lesions (MBP). **(E)** The quantification of positive area of stained markers (*n* = 4 mice per group). **(F)** The mRNA expression levels of *Tnfα*, *Il-1b*, and *Ccr5* in spinal cords from healthy *Nlrx1*^*−/−*^2D2 and spEAE *Nlrx1*^*−/−*^2D2 mice, quantified by qPCR (*n* = 4 or 8 mice per group). **(G)** A representative HE staining of the spinal cords from *Nlrx1*^*−/−*^2D2 spEAE and healthy *Nlrx1*^*−/−*^2D2 mice. **(H)** Immunofluorescence analysis of focal lesions in the spEAE spinal cord and the nuclear localization of NF-κB p65 subunit in CD68^+^ cells (white arrows), 63× magnification. The degree of colocalization was quantified using image J and Pearson correlation coefficient (PCC = 0.6). **(I)** The percentage of CD45^high^, CD45^low^, and CD11b^+^ MHCII^+^ cells in the spinal cord of healthy *Nlrx1*^*−/−*^2D2 and spEAE *Nlrx1*^*−/−*^2D2 mice (*n* = 8 mice per group). **(J)** Representative flow cytometry plots showing the expression of CD11b^+^MHCII^+^ myeloid cells in CD45^+^ cell population. **(K)** An immunofluorescent image of CD3^+^ T cells (shown by white arrows) in the spinal cords of *Nlrx1*^*−/−*^2D2 spEAE mice; magnification 40×; yellow, MBP; blue, DAPI; red, CD3. (**L)** The percentage of Vβ11^+^ T cells in the spinal cord and brain of spEAE *Nlrx1*^*−/−*^2D2 (*n* = 10) compared with healthy *Nlrx1*^*−/−*^2D2 mice (*n* = 7). (**M)** Representative flow cytometric analysis of CD45^+^ Vβ11^+^ T cells in the brain and spinal cord of spEAE and healthy animals. **(N)** The expression of T cell–associated transcription factors in the spinal cord (Sc) and lymph nodes (LN) of *Nlrx1*^*−/−*^2D2 spEAE (*n* = 6) mice compared with *Nlrx1*^*−/−*^2D2 healthy mice (*n* = 5). All data are presented as mean ± SD. *P ≤ 0.05, **P ≤ 0.01, ***P ≤ 0.001, ****P ≤ 0.0001 as determined by the two-tailed Student *t* test. Underlying data can be found in S1 Data. *Ccr5*, C-C chemokine receptor type 5; CNS, central nervous system; GFAP, glial fibrillary acidic protein; HE, hematoxylin–eosin; Iba1, ionized calcium binding adaptor molecule 1; *Il-1b*, interleukin 1 beta; LN, lymph nodes; MBP, myelin basic protein; MHC, major histocompatibility complex; NF-κB, nuclear factor κB; *Nlrx1*, nucleotide-binding, leucine-rich repeat containing X1; PCC, Pearson correlation coefficient; PCR, quantitative polymerase chain reaction; *Rorc*, retinoic acid-related orphan nuclear hormone receptor C; Sc, spinal cord; spEAE, spontaneous EAE; *Tbet*, T-Box transcription factor; *Tnfα*, tumor necrosis factor alpha.

To study what role NLRX1 plays in the initiation of CNS inflammation, we crossed TCR transgenic 2D2 mice to *Nlrx1*^*−/−*^ mice and measured the incidence of spEAE in 2D2 and *Nlrx1*^*−/−*^*2D2* mice housed under the same pathogen-free conditions. We found spEAE in 54% (21 out of 39) of *Nlrx1*^*−/−*^ 2D2 mice, while only 6% (6 out of 95) of 2D2 mice developed spEAE (Fig 1A). The spEAE frequency was similar between males and females (Fig 1B) and the age of onset for the majority of *Nlrx1*^*−/−*^ 2D2 mice was between 6 and 9 weeks (*n* = 16, 76%), while just 24% (*n* = 5) of *Nlrx1*^*−/−*^ 2D2 mice developed spEAE after 9 weeks. The clinical scores rapidly increased from 1.5 to 4 within one week with no signs of recovery, necessitating an early euthanasia of the animals (Fig 1C). The onset of spEAE followed a seasonal pattern. Out of all mice, the most frequent incidence of the disease was in summer (*n* = 11, 53%), followed by spring (*n* = 4, 19%), and the lowest frequency was observed in fall and winter (*n* = 3, 14%). The observed neurological symptoms are similar to the symptoms that develop during the classical immunization-induced EAE. Therefore, we hypothesized that spEAE animals would present pathophysiological changes within the CNS similar to those found in classical EAE.

When we assessed the presence of inflammation in the CNS of *Nlrx1*^*−/−*^ 2D2 spEAE mice, we found that the lack of NLRX1 did not affect the expression of glial fibrillary acidic protein (GFAP), ionized calcium binding adaptor molecule 1(Iba1), and myelin basic protein (MBP) in the spinal cords of healthy *Nlrx1*^*−/−*^ 2D2 mice (S1A Fig). We did, however, find that the expression of Iba1 and GFAP (microglial and astrocytic markers) as well as the inflammatory mediators tumor necrosis factor alpha (*Tnfα*), interleukin 1 beta (*Il-1β*), and C-C chemokine receptor type 5 (*Ccr5*) was significantly increased in *Nlrx1*^*−/−*^2D2 spEAE spinal cords, while the expression of MBP was significantly decreased compared with healthy *Nlrx1*^*−/−*^ 2D2 mice (Fig 1D–1F).

Because many studies reported that NLRX1 can inhibit NF-κB and Type I interferon signaling pathways, we measured the expression of Type I interferons in the brains of *Nlrx1*^*−/−*^ 2D2 mice and found that the expression of interferon beta (IFNβ) was significantly increased in spEAE mice compared with *Nlrx1*^*−/−*^2D2 healthy mice (S1B Fig). Additionally, hematoxylin staining demonstrated a massive infiltration of inflammatory cells into the spinal cord of *Nlrx1*^*−/−*^2D2 spEAE animals (Fig 1G), in which nuclear localization of NF-κB p65 subunit was observed (S1C Fig). Immunofluorescence analysis of focal lesions revealed the nuclear localization of NF-κB p65 subunit in CD68^+^ cells (Fig 1H, S2A Fig) but not in GFAP^+^ astrocytes (S2B Fig). Furthermore, flow cytometry analysis showed significant increases in accumulation of peripheral immune cells (CD45^high^), including a greater percentage of activated CD11b^+^ MHCII^+^ myeloid cells in the spinal cord of *Nlrx1*^*−/−*^2D2 spEAE mice compared with *Nlrx1*^*−/−*^2D2 healthy mice (Fig 1I and 1J). Moreover, the spinal cords of *Nlrx1*^*−/−*^*2D2* spEAE mice showed a marked infiltration of myelin-specific transgenic TCR (Vβ11^+^) T cells into the CNS (Fig 1K–1M) and enhanced expression of T helper (Th)1–associated transcription factor, T-Box transcription factor (*Tbet*), in the spinal cords of *Nlrx1*^*−/−*^ 2D2 spEAE mice (Fig 1N). Although there were no differences in the percentages of Vβ11^+^ T cells (S3A Fig) and the expression of T-cell activation markers (CD44, CD25) in the spleen (S3B Fig), we found an increased expression of *Tbet* and IL-17 in the lymph nodes of *Nlrx1*^*−/−*^*2D2* spEAE mice compared with *Nlrx1*^*−/−*^*2D2* healthy mice (Fig 1N and S3C Fig).

To determine the T cell–intrinsic activity of *Nlrx1*, we compared the activation of CD4^+^ T cells from naïve 2D2 and *Nlrx1*^*−/−*^ 2D2 mice using myelin oligodendrocyte glycoprotein (MOG)-pulsed WT and *Nlrx1*^*−/−*^ splenocytes. We did not observe any difference between the capability of WT and *Nlrx1*^*−/−*^ splenocytes as antigen presenting cells (APCs) in activating T cells in vitro (S4A–S4D Fig); however, we found significant increases in the proliferation of *Nlrx1*^*−/−*^2D2 T cells compared with 2D2 T cells, confirmed by ^3^H-thymidine incorporation assay and Ki67 staining (S5A–S5E Fig). We also observed elevated production of interferon (IFN)γ by activated *Nlrx1*^*−/−*^ 2D2 T cells (S5F Fig and S5G Fig) and a differentiation bias toward Th1 and Th17 cells (S5H Fig and S5I Fig).

Immunoblotting and immunofluorescence tests revealed significant increases of immunoglobulin G (IgG) in the spinal cords of *Nlrx1*^*−/−*^2D2 spEAE mice compared with healthy controls (S6A Fig–S6C Fig). The flow cytometry analysis of inflamed spinal cord tissue demonstrated significant increases in the number of CD19^+^ B cells and serum levels of anti-MOG antibody in *Nlrx1*^*−/−*^2D2 spEAE animals (S6D Fig–S6F Fig). The analysis of pathophysiological changes in spEAE *Nlrx1*^*−/−*^2D2 mice suggested the development of a severe form of CNS inflammation compatible with the classical form of EAE associated with astrogliosis, microgliosis, Th1 and Th17 bias, increased expression of pro-inflammatory proteins, and demyelination. Next, we compared the magnitude and the severity of CNS inflammation between *Nlrx1*^*−/−*^2D2 spEAE and 2D2 spEAE mice.

### *Nlrx1*^*−/−*^ 2D2 mice develop more severe EAE than 2D2 mice

**Fig 2:**
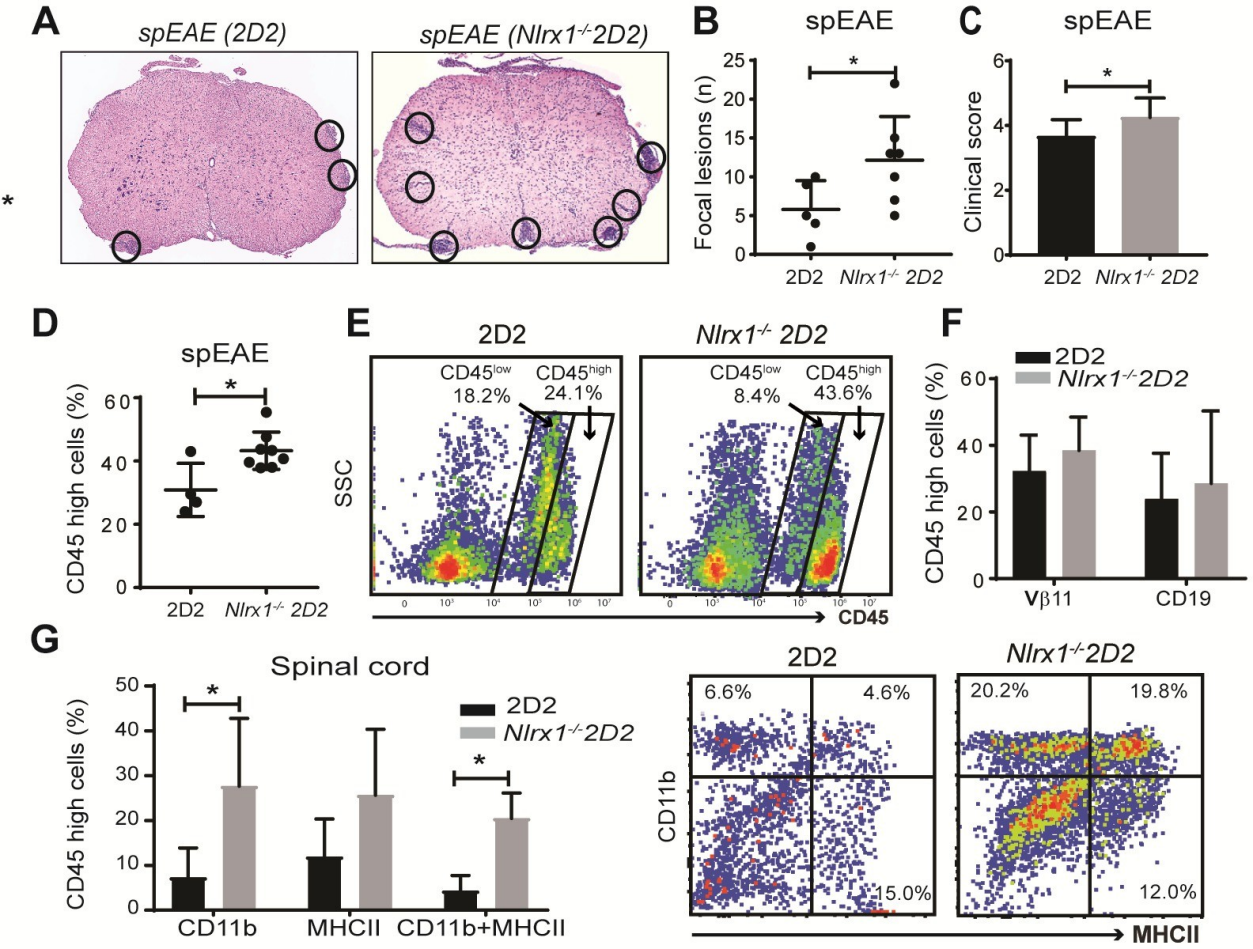
CNS inflammation associated with spEAE is more severe in *Nlrx1*^*−/−*^2D2 compared with 2D2 mice. **(A)** Representative image of HE staining of lumbar spinal cords from spEAE mice with *Nlrx1*^*−/−*^2D2 or 2D2 genotype. The circles show focal lesions. **(B)** The number of focal lesions counted in spinal cords from spEAE mice (*n* = 5, 7 in each group). **(C)** EAE clinical score in *Nlrx1*^*−/−*^ *2D2* (*n* = 18) or 2D2 (*n* = 6) mice at the time of euthanization. **(D)** The percentage of CD45^high^ cells in the spinal cords of *Nlrx1*^*−/−*^2D2 spEAE (*n* = 6) mice compared with 2D2 spEAE mice (*n* = 4). **(E)** The flow cytometry plots of CD45^high^ gate. **(F)** Similar percentages of Vβ11^+^ T cells and CD19^+^ B cells in the spinal cords of spEAE mice (*n* = 5, 7). **(G)** Elevated numbers of activated CD11b^+^MHCII^+^ monocyte/macrophage in CD45^high^ cell population, quantified in spinal cords from *Nlrx1*^*−/−*^2D2 spEAE (*n* = 6) or 2D2 spEAE (*n* = 4) mice by flow cytometry. Data are presented as mean ± SD. *P ≤ 0.05 determined by the two-tailed Student *t* test. Underlying data can be found in S1 Data. CNS, central nervous system; EAE, experimental autoimmune encephalomyelitis; HE, hematoxylin–eosin; MHC, major histocompatibility complex; *Nlrx1*, nucleotide-binding, leucine-rich repeat containing X1; spEAE, spontaneous EAE.

Because we found a higher incidence of spEAE in *Nlrx1*^*−/−*^2D2 mice compared with 2D2 mice, we hypothesized that *Nlrx1*^*−/−*^2D2 mice develop a greater CNS inflammation and more severe spEAE than 2D2 mice. Indeed, we observed significantly higher clinical scores, increased numbers of focal lesions, and a higher percentage of activated CD11b^+^MHCII^+^ cells in the spinal cords of affected *Nlrx1*^*−/−*^ 2D2 compared with affected 2D2 mice. However, no difference was found between the percentage of lymphoid cells, including myelin-specific Vβ11^+^ T cells and CD19^+^ B cells in the spinal cords of spEAE mice from both genotypes (Fig 2A–2G). These findings led us to investigate the immunoregulatory role of NLRX1 in innate immune cells beyond antigen presentation and T-cell activation. For these experiments we took advantage of lymphocyte-deficient recombination-activating gene (*Rag*)^*−/−*^ mice.

### NLRX1 inhibits innate immune response and prevents CNS inflammation

**Fig 3:**
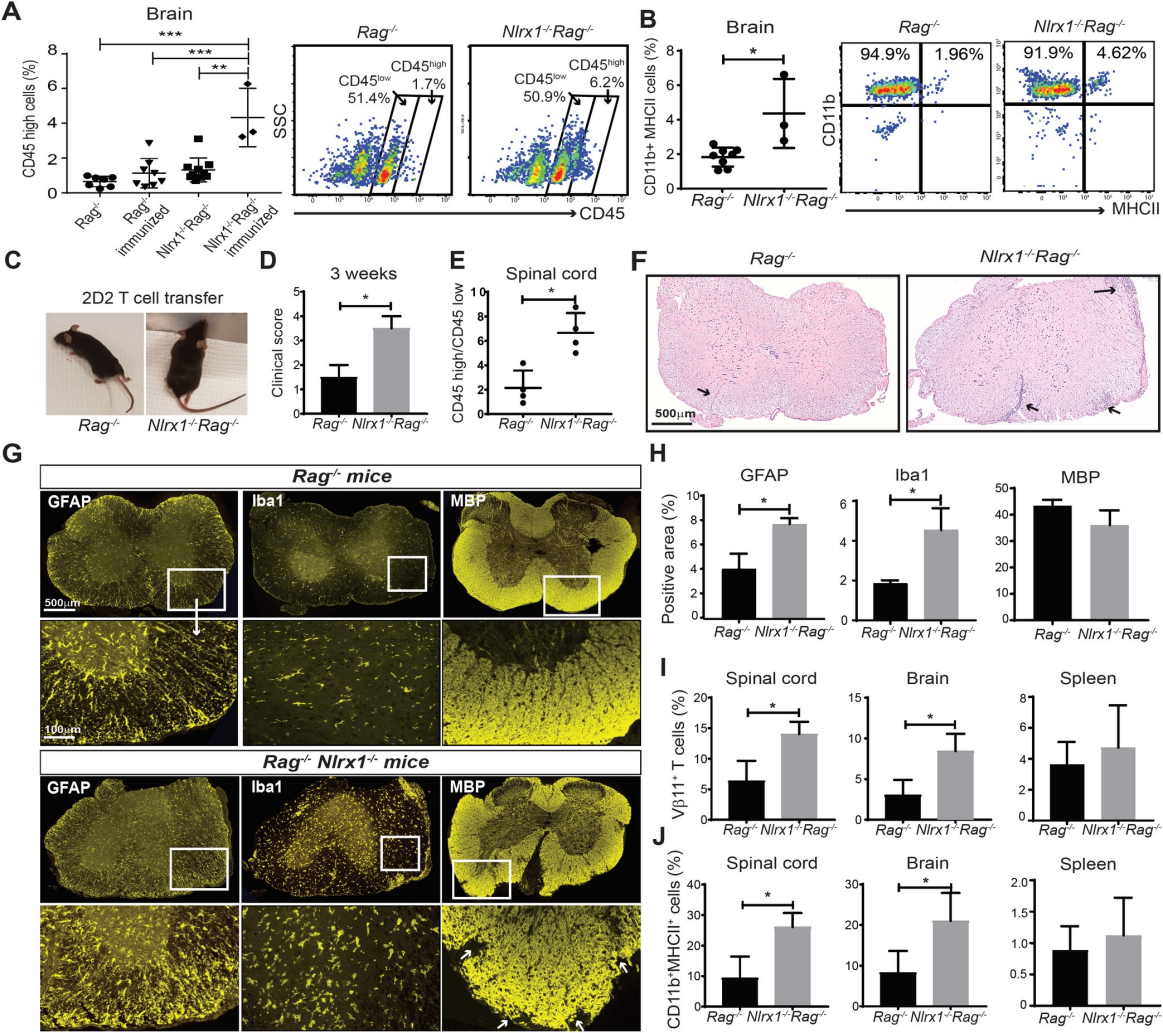
Activation of innate immune cells induces CNS inflammation and severe paralysis in *Nlrx1*^*−/−*^ *Rag*^*−/−*^ mice. **(A)** The infiltration of CD45^high^ leukocytes to the brain of *Nlrx1*^*−/−*^*Rag*^*−/−*^ mice (*n* = 3) compared with *Rag*^*−/−*^ mice (*n* = 5) 14 days after immunization with MOG-CFA emulsion plus PTX, quantified by flow cytometry as shown in representative plots, ANOVA test. **(B)** The percentage of activated CD11b^+^MHCII^+^ microglia/macrophages in CD45^+^ cells, quantified by flow cytometry as shown in representative plots, Mann–Whitney *U* test. (**C)** Adoptive transfer of 2D2 T cells followed by MOG-CFA/PTX immunization caused hind limb paralysis in *Nlrx1*^*−/−*^*Rag*^*−/−*^ mice, which was not observed in *Rag*^*−/−*^. **(D)** The clinical score of mice 3 weeks after adoptive transfer and immunization (*n* = 4 mice per group). **(E)** The ratio of CD45^high^ cells (myeloid cells) to CD45^Low^ cells (microglia) in the spinal cords of *Nlrx1*^*−/−*^*Rag*^*−/−*^ mice compared with *Rag*^*−/−*^ mice following adoptive T-cell transfer and MOG-CFA/PTX immunization (*n* = 4 mice per group). **(F)** The HE staining of spinal cords from *Nlrx1*^*−/−*^*Rag*^*−/−*^ mice and *Rag*^*−/−*^ mice, black arrows show the infiltration of mononuclear cells, magnification 40×. (**G)** The expression of GFAP, Iba1, and MBP in the spinal cords of *Nlrx1*^*−/−*^*Rag*^*−/−*^ mice compared with *Rag*^*−/−*^ mice, magnification 40×. **(H)** Quantification of stained markers (Iba1, microglia; GFAP, astrocyte; MBP, myelin basic protein) using Image J software (*n* = 3 mice per group). **(I)** The percentage of Vβ11^+^ T cells in the spinal cord, brain, and spleen of *Nlrx1*^*−/−*^*Rag*^*−/−*^ mice compared with *Rag*^*−/−*^ mice (*n* = 4 mice per group). **(J)** The percentage of activated CD11b^+^MHCII^+^ microglia/macrophage in the spinal cords, brains, and spleens of *Nlrx1*^*−/−*^*Rag*^*−/−*^ mice compared with *Rag*^*−/−*^ mice (*n* = 4 mice per group). All the data are presented as mean ± SD. *P ≤ 0.05, **P ≤ 0.01, ***P ≤ 0.001 determined by the two-tailed Student *t* test, except for the data that mentioned one-way ANOVA with Tukey post hoc or Mann–Whitney *U* test. Underlying data can be found in S1 Data. CFA, Complete Freund's Adjuvant; CNS, central nervous system; GFAP, glial fibrillary acidic protein; HE, hematoxylin–eosin; Iba1, ionized calcium binding adaptor molecule 1; MBP, myelin basic protein; MHC, major histocompatibility complex; MOG, myelin oligodendrocyte glycoprotein; *Nlrx1*, nucleotide-binding, leucine-rich repeat containing X1; PTX, pertussis toxin; *Rag*, recombination-activating gene.

First, to test the effect of NLRX1 on CNS inflammation in the absence of adaptive immunity, we immunized *Rag*^*−/−*^ and *Nlrx1*^*−/−*^*Rag*^*−/−*^ mice with MOG-Complete Freund's Adjuvant (CFA) emulsion plus pertussis toxin (PTX) and quantified the percentage of myeloid cells involved in the CNS innate immune response on day 14 postimmunization. Flow cytometry data showed significant increases in the percentages of CD45^high^ leukocytes in the brains and spinal cords of *Nlrx1*^*−/−*^*Rag*^*−/−*^ mice compared with *Rag*^*−/−*^ mice after immunization (Fig 3A and S7A Fig–S7D Fig). We also found increased percentages of activated CD11b^+^MHCII^+^ myeloid cells in the brains of *Nlrx1*^*−/−*^*Rag*^*−/−*^ mice compared with *Rag*^*−/−*^ mice after MOG-CFA/PTX immunization (Fig 3B).

Second, we reintroduced T cells into the experimental paradigm and investigated whether *Nlrx1*^*−/−*^*Rag*^*−/−*^ mice are more susceptible to EAE than *Rag*^*−/−*^ mice after adoptive transfer of myelin-specific T cells. We transferred MOG-specific 2D2 T cells into *Rag*^*−/−*^ and *Nlrx1*^*−/−*^ *Rag*^*−/−*^ mice and following 21 days of immunization with MOG-CFA emulsion plus PTX, we observed several changes. We found severe hind limb paralysis, higher EAE clinical score (3.5 ± 0.7 versus 1.5 ± 0.7), and increased accumulation of CD45^high^ cells in *Nlrx1*^*−/−*^*Rag*^*−/−*^ compared with *Rag*^*−/−*^ mice (Fig 3C–3E). Moreover, we found inflammatory foci, demyelinating lesions, and elevated Iba1 and GFAP expression in the spinal cords of *Nlrx1*^*−/−*^*Rag*^*−/−*^ mice (Fig 3F–3H). Additionally, the percentages of myelin-specific T cells and CD11b^+^ MHCII^+^ myeloid cells were significantly increased in the CNS tissues from *Nlrx1*^*−/−*^*Rag*^*−/−*^ mice compared with *Rag*^*−/−*^ mice (Fig 3I and 3J). No significant difference was found in the percentage of myelin-specific T cells and CD11b^+^ MHCII^+^ myeloid cells in the spleens of *Nlrx1*^*−/−*^*Rag*^*−/−*^ mice compared with *Rag*^*−/−*^ mice (Fig 3I and 3J). The mRNA levels of T-cell activation markers (CD44, CD25) and inflammatory T-cell cytokines (IFNγ, IL-17) were similar in the spleens of *Rag*^*−/−*^ mice and *Nlrx1*^*−/−*^*Rag*^*−/−*^mice (S7E Fig). From these experiments, although we cannot exclude the effect of NLRX1 in shaping the adaptive immune response in EAE, we can conclude that NLRX1 plays an immunoregulatory role in the innate immune compartment.

**Fig 4:**
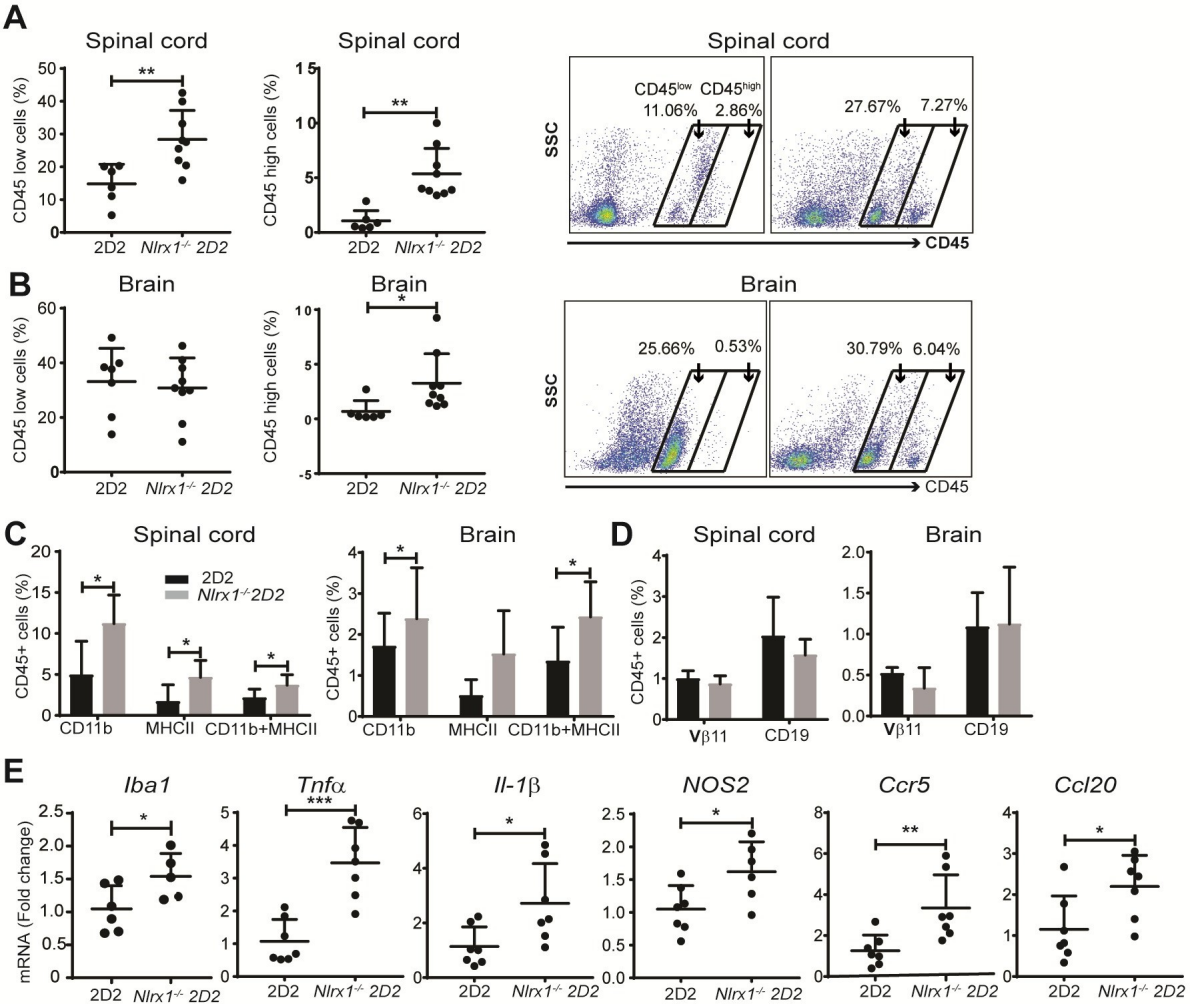
Preclinical stages of inflammation in the CNS of *Nlrx1*^*−/−*^2D2 mice. **(A)** The percentages of CD45^low^ microglia and CD45^high^ myeloid cells in the spinal cords and brains of *Nlrx1*^*−/−*^2D2 mice compared with 2D2 mice, quantified by flow cytometry, Mann–Whitney *U* test. (**B)** Representative plots showing CD45^low^ and CD45^high^ gates in spinal cord and brain samples from *Nlrx1*^*−/−*^2D2 and 2D2 mice, Mann–Whitney *U* test. **(C)** The percentage of activated myeloid cells (CD11b^+^MHCII^+^) in the spinal cord and brain of *Nlrx1*^*−/−*^2D2 and 2D2 mice (*n* = 8 mice per group). **(D)** The percentage of Vβ11^+^ T cells and CD19^+^ B cells in the spinal cords and brains of *Nlrx1*^*−/−*^2D2 and 2D2 mice, quantified by flow cytometry (*n* = 6 mice per group). **(E)** The mRNA levels of Iba1 and inflammatory mediators in *Nlrx1*^*−/−*^2D2 brains compared with 2D2 brains, quantified by qPCR (*n* = 7 mice per group). All the data are presented as mean ± SD. *P ≤ 0.05, **P ≤ 0.01, ***P ≤ 0.001 as determined by the Student *t* test, except when Mann-Whitney *U* test was specified. Underlying data can be found in S1 Data. CNS, central nervous system; Iba1, ionized calcium binding adaptor molecule 1; MHC, major histocompatibility complex; *Nlrx1*, nucleotide-binding, leucine-rich repeat containing X1; qPCR, quantitative polymerase chain reaction.

The previous experiments point to a role of NLRX1 in the innate immune response. Therefore, we hypothesized that *Nlrx1*^*−/−*^ 2D2 mice would have a higher level of activated innate immune cells in the CNS, even in the absence of symptoms. Accordingly, we evaluated the activation status of infiltrating innate immune cells and microglia in the CNS of asymptomatic *Nlrx1*^*−/−*^2D2 and 2D2 mice by flow cytometry. Although quantification of immunofluorescence of Iba1-positive cells did not reveal any statistically significant changes (S1A Fig), at the subclinical stage, we observed significantly higher percentages of CD45^low^ microglia in the spinal cords of *Nlrx1*^*−/−*^2D2 mice, while the percentage of CD45^high^ cells was significantly increased in both brains and spinal cord tissues from *Nlrx1*^*−/−*^ 2D2 mice compared with 2D2 mice (Fig 4A and 4B). We found increased percentages of the CD11b^+^ MHCII^+^ population in the CNS tissues of asymptomatic *Nlrx1*^*−/−*^ 2D2 mice compared with 2D2 mice (Fig 4C), while the percentages of T cells and B cells were comparable in both genotypes (Fig 4D). Additionally, we found a marked increase in the mRNA expression of Iba1 as well as the inflammatory mediators *Tnfa*, *Il-1β*, *Ccl20*, *Ccr5*, and nitric oxide synthase (*NOS2*) in the brains of *Nlrx1*^*−/−*^ 2D2 mice compared with 2D2 mice (Fig 4E). The pro-inflammatory molecules that we measured are often associated with increased migration, generation of neurotoxic glia, and cell death [24–27]. Altogether, these results suggest that there is an increased myeloid cell activation in the CNS of *Nlrx1*^*−/−*^ mice without the presence of lymphocytes. Next, we investigated whether the inflammatory milieu within the CNS is associated with cell death and tissue damage.

### NLRX1 inhibits tissue damage and the generation of neurotoxic astrocytes

**Fig 5:**
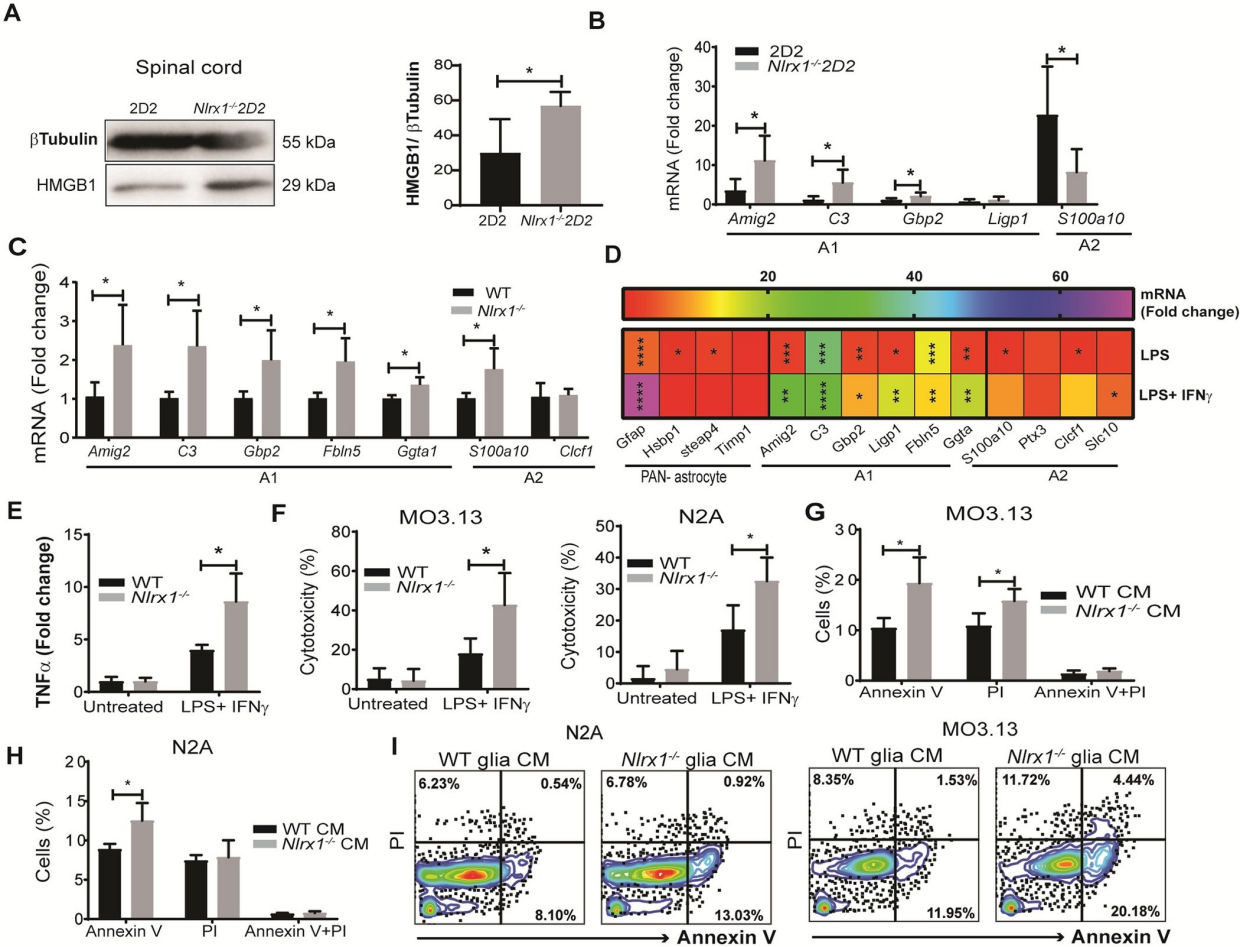
NLRX1 inhibits the tissue damage and generation of neurotoxic glia in the CNS at the subclinical stage of EAE. **(A)** The level of HMGB1 in the spinal cord of asymptomatic *Nlrx1*^*−/−*^2D2 mice compared with 2D2 mice, determined using western blot and quantified by the percentage of HMGB1 to β-tubulin ratio (*n* = 6 mice per group). **(B)** The levels of A1- and A2-related gene expression in brain from *Nlrx1*^*−/−*^2D2 mice compared with 2D2 mice (*n* = 5 mice per group). **(C)** The expression of A1-related genes in the brains of *Nlrx1*^*−/−*^ *m*ice compared with WT mice (*n* = 5). **(D)** A heatmap diagram showing the fold change of mRNA levels of pan-, A1- or A2-reactive astrocyte-related genes in *Nlrx1*^*−/−*^ glia culture after 24 hours of stimulation with LPS (500 ng/mL) or LPS+IFNγ (10 ng/mL) compared with the corresponding WT controls (*n* = 4 independent samples in each group). **(E)** TNFα level in the conditioned medium (CM), collected 24 hours after LPS/IFNγ treatment of glia culture and measured by ELISA (*n* = 4 independent samples/group). **(F)** The toxicity of *Nlrx1*^*−/−*^ or WT glia CM on MO3.13 and N2A cells after 24 hours, measured by MTT assay (*n* = 6 independent samples/group). **(G)** Expression of apoptosis (annexin V) and permeability (propidium iodide [PI]) markers on MO3.13 cells incubated with *Nlrx1*^*−/−*^glia CM compared with WT glia CM for 24 hours (*n* = 4 independent samples/group). **(H)** The percentage of annexin V–and PI-positive N2A cells incubated with *Nlrx1*^*−/−*^glia CM compared with WT glia CM for 24 hours (*n* = 4 independent samples/group). **(I)** Representative flow cytometry plots used for the quantification of annexin V and PI positivity of MO3.13 and N2A cells. All the data are presented in mean ± SD. *P ≤ 0.05, **P ≤ 0.01, ***P ≤ 0.001, determined by the two-tailed Student *t* test. Underlying data can be found in S1 Data. CM, conditioned medium; CNS, central nervous system; EAE, experimental autoimmune encephalomyelitis; HMGB1, high mobility group box 1; IFNγ, interferon gamma; LPS, lipopolysaccharide; MTT, 3-(4,5-Dimethylthiazol-2-yl)-2,5-Diphenyltetrazolium Bromide; NLRX1, nucleotide-binding, leucine-rich repeat containing X1; PI, propidium iodide; TNFα, tumor necrosis factor alpha; WT, wild-type.

Because we found that the expression of potentially harmful molecules such as TNFα, inducible nitric oxide synthase (iNOS), and IL-1β is elevated in *Nlrx1*^*−/−*^ mice, we hypothesized that there is an increase in neurotoxic glia and exacerbated tissue injury in *Nlrx1*^*−/−*^ 2D2 mice. Consistent with this hypothesis, we observed a significantly higher degree of tissue injury in the CNS of *Nlrx1*^*−/−*^ 2D2 mice compared with 2D2 mice, as indicated by the levels of high mobility group box 1 (HMGB1) in the spinal cord (Fig 5A). The tissue injury was associated with significantly higher expression of A1-related reactive astrocyte genes, while the level of the A2-related astrocyte gene, s100a10, was significantly lower compared with 2D2 mice (Fig 5B). We observed a similar pattern of higher expression of A1-related genes in *Nlrx1*^*−/−*^ brains compared with WT brains (Fig 5C).

To better understand the role of NLRX1 in astrocyte phenotypes, we stimulated standard glial cultures from *Nlrx1*^*−/−*^ and WT mice with lipopolysaccharide (LPS)/IFNγ and found that *Nlrx1*^*−/−*^ glia express significantly increased levels of A1-related transcripts and had a higher level of TNFα production than WT glia (Fig 5D and 5E). We further investigated the cytotoxic effect of conditioned medium collected from LPS/IFNγ-treated glial cells on neurons and oligodendrocytes. The results showed a significantly lower rate of survival and higher rate of death in both N2A and MO3.13 cells treated with *Nlrx1*^*−/−*^ glia compared with WT glia-conditioned medium (Fig 5F–5H).

### *NLRX1* expression analysis in MS patients and EAE mice

**Fig 6:**
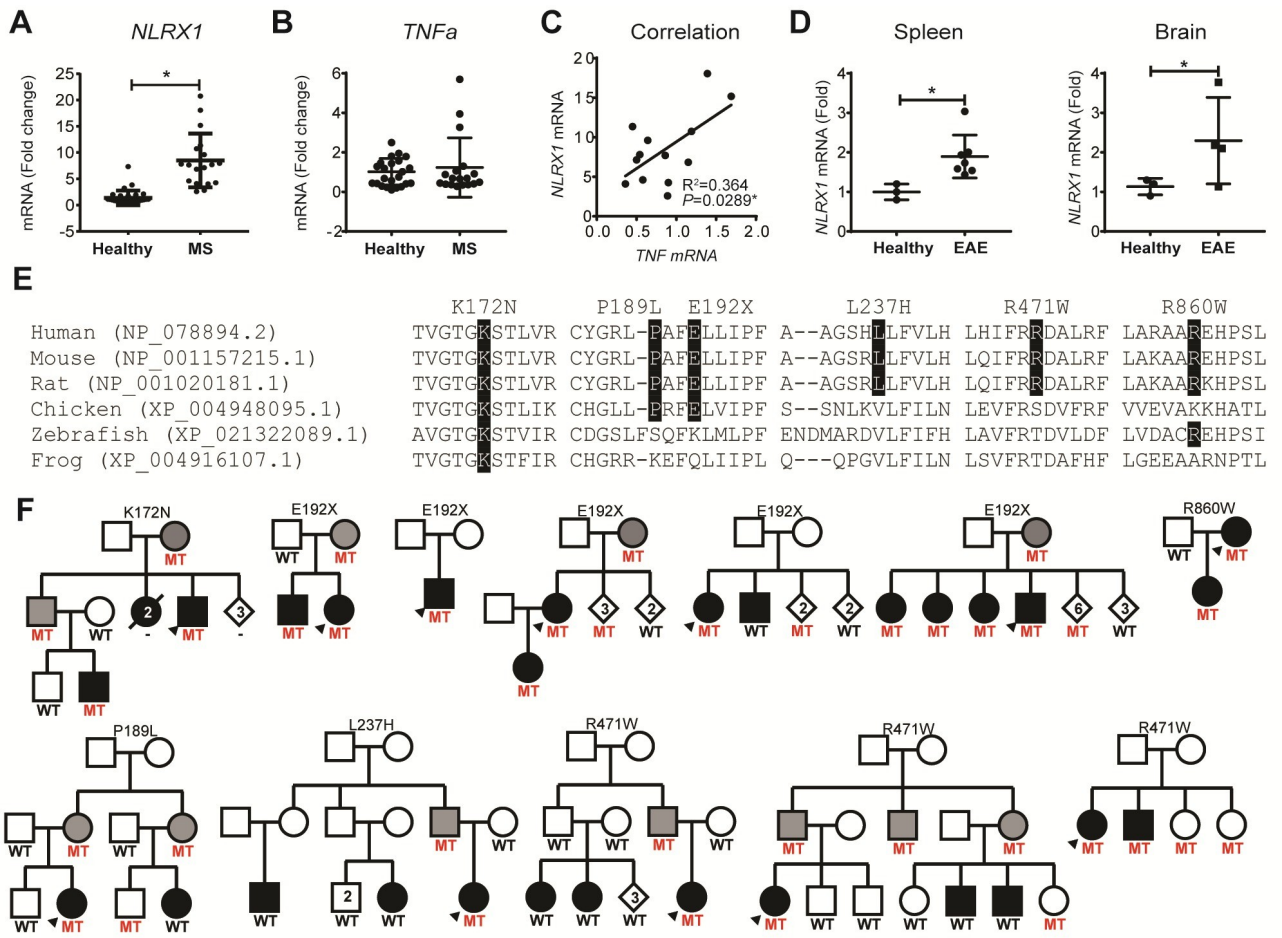
NLRX1: Implications for human MS. **(A)** Expression of *NLRX1* in PBMC from MS patients (*n* = 18) compared with healthy donors (*n* = 31), quantified using qPCR. The data are presented as mean ± SD; *P ≤ 0.05, determined by two-tailed Student *t* test. **(B)** The mRNA levels of *TNFα* expression in PBMC from MS patients (*n* = 18) and healthy individuals (*n* = 28), measured by qPCR. **(C)** Correlation between mRNA levels of *NLRX1* and *TNFα* in the PBMC from MS samples (*n* = 13), statistically determined using Pearson correlation coefficient, *r* = 0.604, *P = 0.029. **(D)** Expression of *Nlrx1* in the spleen and brain of C57BL/6J mice with classical EAE compared with healthy mice. The WT mice were immunized with MOG/CFA and pertussis. The brain and spleen were collected 3 weeks after immunization; *P ≤ 0.05, determined by two-tailed Student *t* test. Underlying data can be found in S1 Data. **(E)** *NLRX1* conservation in orthologs. Evolutionarily conserved positions for the identified mutations are highlighted in black. Organism and RefSeq accession numbers are provided. **(F)** Pedigrees for families identified with *NLRX1* mutations. Black filled symbol, MS; gray filled, unaffected obligate carrier. Heterozygote mutation carriers (MT) and WT genotypes are indicated. CFA, Complete Freund's Adjuvant; EAE, experimental autoimmune encephalomyelitis; MOG, myelin oligodendrocyte glycoprotein; MS, multiple sclerosis; MT, mutation carrier; NLRX1, nucleotide-binding, leucine-rich repeat containing X1; PBMC, peripheral blood mononuclear cell; qPCR, quantitative polymerase chain reaction; *TNFα*, tumor necrosis factor alpha; WT, wild-type.

Our next question was whether the expression level of *Nlrx1* would change in CNS inflammation. During inflammation, the adaptive and innate immune responses in the CNS are often reflected in the peripheral blood [28,29]. Therefore, sampling the peripheral blood may provide an understanding on the processes that occur within the CNS. We quantified the expression of *NLRX1* in peripheral blood mononuclear cells (PBMCs) from MS patients (S2 Table) and found that PBMCs from relapsing-remitting MS (RRMS) patients express significantly higher levels of *NLRX1* mRNA than healthy controls (Fig 6A). We found an increased expression of *NLRX1* mRNA in purified CD14^+^ myeloid cells compared with CD3^+^ T cells in patients’ PBMCs (S8 Fig). We also compared the expression of *TNFα* in PBMCs from the RRMS patients and healthy controls and found no difference. However, there was a significant positive correlation between the mRNA levels of *NLRX1* and *TNFα* in the PBMCs from RRMS patients (Fig 6B and 6C). Similarly, in mice, we found a significant increase in the mRNA levels of *Nlrx1* in spleen and brain tissues from EAE mice compared with healthy mice (Fig 6D), suggesting that NLRX1 may be a part of a negative feedback loop triggered by inflammation. In mouse EAE, the increased expression of *Nlrx1* is caused by high doses of immunization that are designed to overcome all endogenous inhibitors of inflammation. In patients, however, the disease develops over years and presents as an outcome of pro-inflammatory molecules overcoming the endogenous anti-inflammatory pathways. This process can be associated with genetic and environmental factors [30].

### Assessment of *NLRX1* genetic variants in MS patients

To determine whether *NLRX1* genetic variants are implicated in the onset of MS in humans, we mined exome sequencing data from 326 MS patients and 123 healthy controls. Variants identified exclusively in MS patients, with a minor allele frequency below 1% in publicly available databases (GnomAD) [31], and resulting in missense or nonsense substitutions, were considered potentially disease relevant. This analysis identified five missense mutations (p.Lys172Asn, p.Pro189Leu, p.Leu237His, p.Arg471Trp, and p.Arg860Trp), each in one patient; and one nonsense mutation (p.Glu192Ter) in three MS patients (S3 Table). All mutations are predicted to be damaging on protein function, with Combined Annotation Dependent Depletion (CADD) phred-scale scores ranging from 22 to 35 [32], and affected amino acids are evolutionarily conserved in mammals (Fig 6E). In addition, the NLRX1 p.Lys172Asn mutation, which is located in the NATCH domain, appears to also be conserved in vertebrates and all human paralogs (NLRP1 to NLRP14; S9 Fig).

To further define the role of these variants in MS, we genotyped *NLRX1* variants in a case-control series from Canada and only identified p.Arg471Trp in two additional patients and two controls and p.Glu192Ter in two additional patients and one control. Segregation analysis within families did not support cosegregation with MS for p.Pro189Leu, p.Leu237His, and p.Arg471Trp, with less than 75% of affected family members harboring the mutations (Fig 6F); therefore, these three variants are unlikely to play a role in the onset of MS. In contrast, NLRX1 p.Lys172Asn was only observed in two affected individuals and two obligate carriers from one family, and p.Arg860Trp was only present in a mother and daughter, both diagnosed with MS, thus suggesting a role in disease. The nonsense mutation, p.Glu192Ter, was identified in four multi-incident families and one patient without a family history of MS. In these families, the majority of individuals diagnosed with MS were found to carry the p.Glu192Ter mutation (10/11); however, 14 healthy individuals, including three obligate carriers, were also found to harbor this mutation, suggesting that patients harboring this truncating mutation have a 41.6% (10/24) chance of developing MS, despite its reduced penetrance.

## Discussion

This work provides the first evidence of the potential role of an innate immune receptor, NLRX1, in predisposition to EAE. We generated *Nlrx1*^*−/−*^ 2D2 mice that are 10 times more likely to develop spEAE than 2D2 mice and have increased CNS tissue inflammation, indicating that NLRX1 prevents the onset of EAE. Although NLRX1 inhibits both innate and adaptive immune responses, the expression of NLRX1 in the innate immune compartment suppresses the development of spontaneous disease. Mechanistically, we demonstrate that in the absence of NLRX1, astrocytes exposed to pro-inflammatory conditions have elevated levels of A1-associated transcripts and lower levels of A2-associated transcripts. Stimulated *Nlrx1*^*−/−*^ mixed-glia-conditioned media is more toxic to neuronal and oligodendrocyte cell lines. Importantly, we found that NLRX1 expression was elevated in the peripheral immune cells of MS patients and EAE mice relative to their respective controls. This is further supported by the identification of six rare *NLRX1* mutations in MS patients, including a p.Glu192Ter truncation in 10 patients.

In agreement with work by Eitas and colleagues that showed NLRX1 inhibits the progression of EAE following immunization with MOG-CFA [11], we demonstrated that NLRX1 reduces the severity of spEAE. Furthermore, our results suggest that NLRX1 plays a role in tissue inflammation prior to the appearance of clinical symptoms. Thus, NLRX1 both inhibits onset of EAE and reduces its severity, which explains why *Nlrx1*^*−/−*^2D2 animals develop a rapidly progressing EAE with no recovery. A similarly acute progressive EAE course has been previously reported in TCR transgenic mice in the Swiss Jim Lambert (SJL) background and *Tnfr2*^*−/−*^2D2 female mice [33,34].

Although there are many TCR transgenic models of EAE (S1 Table), our study is the first to demonstrate the crucial role of a PRR in the etiology of spEAE. We provide evidence that expression of NLRX1 in the innate immune compartment is sufficient to suppress T cell–mediated autoimmunity. Furthermore, we observed a seasonal pattern in the onset of spEAE, as we found the highest frequency of *Nlrx1*^*−/−*^ 2D2 spEAE in the summer, suggesting that environmental factors influence the disease onset. Although the nature of such fluctuations is unknown, these results are in agreement with studies that have found seasonal changes in disease activity in MS patients [35] and in animal models [36]. To our knowledge, this is the first time that seasonality is reported in a TCR transgenic mouse model of MS.

Pathophysiology of the spEAE in *Nlrx1*^*−/−*^2D2 mice is consistent with the autoimmune nature of the disease. Histopathological examination revealed the massive inflammation and demyelination in the spinal cord of *Nlrx1*^*−/−*^2D2 mice. Activated inflammatory myeloid cells, myelin-specific Tbet^+^ T cells, and IgG deposits in the spinal cord of *Nlrx1*^*−/−*^2D2 mice suggest Th1-mediated autoimmune bias. These findings parallel other mouse models of spEAE [37,38] For example, it was shown that *Tbet*^*−/−*^2D2 mice are protected from EAE [37].

In comparison with the occasional cases of spEAE in 2D2 mice, we found *Nlrx1*^*−/−*^2D2 spEAE mice had a higher EAE clinical score, which was associated with increased numbers of focal lesions. Although the percentages of myelin-specific T cells in the spinal cord of spEAE animals from both genotypes are similar, the differentiation of CD4^+^ T cells toward the inflammatory Th1 or Th17 cells is enhanced in *Nlrx1*^*−/−*^ 2D2 spEAE mice. Interestingly, this effect is T-cell autonomous because it does not depend on the context of APCs. Consistent with our findings, a previous study by Leber and colleagues reported the greater proliferation rates and the higher ability of *Nlrx1*^*−/−*^ T cells to differentiate into the Th17 phenotype when the cells were activated by a nonspecific stimulator, anti-CD3 and anti-CD28, in vitro [39]. However, NLRX1 had no effect on the differentiation of T cells to regulatory T (Treg) cells [39].

Our findings and previous research [11] suggest that NLRX1 inhibits initiation and progression of EAE on at least two levels. At the level of innate immunity, it inhibits activation of microglia and macrophages, and at the level of adaptive immunity, NLRX1 inhibits activation and proliferation of encephalitogenic T-cell phenotypes.

To differentiate between the role of NLRX1 in innate and adaptive immune responses, we crossed the *Nlrx1^-/-^* mice with *Rag^-/-^* mice and found that after adoptive transfer of activated T cells, *Nlrx1*^*−/−*^ *Rag*^*−/−*^ mice developed more severe EAE compared with *Rag*^*−/−*^ mice. Interestingly, even in the absence of T cells, after immunization with CFA, we found greater infiltration of CD45 and CD11b myeloid cells into the CNS of *Nlrx1*^*−/−*^*Rag*^*−/−*^ mice.

Consistent with our findings, Soulika and colleagues reported that the mRNA levels of CD45, CD11b, and *Il-1β* in the spinal cords of *Rag*^*−/−*^ mice were significantly induced after 7 days of MOG-CFA injection [40], indicating that the activation of CNS innate cells begins prior to substantial accumulation of peripheral immune cells in the CNS. Interestingly, Ajami and colleagues suggested that the initial activation of innate immune cells may depend on influx of peripheral monocytes that do not contribute to the microglial pool later in the disease [41].

Furthermore, we found an increased percentage of CD45^low^ microglia and CD45^high^ macrophages in the CNS of asymptomatic *Nlrx1*^*−/−*^2D2 mice compared with healthy 2D2 mice, while the percentages of infiltrating lymphocytes remained very low. We found significant increases in the levels of multiple pro-inflammatory mediators, including *Tnf*α, *NOS2*, *Ccr5*, and *Ccl20* at the subclinical stage in *Nlrx1*^*−/−*^ 2D2. Martin Blondel and colleagues showed that CCR5 is widely expressed on APCs, such as macrophages and microglia, as well as effector T cells [42], whereas CCL20 mainly derived from TNFα-activated astrocytes and functions as a chemoattractant for recruiting CCR6-expressing Th17 cells to the brain [43]. In vitro studies show that IL-1β induces the production of CCL20 in astrocytes, leading to blood-brain barrier (BBB) disruption and ultimately promoting the influx of inflammatory cells [44,45]. We also found an enhanced level of HMGB1 in the spinal cord of *Nlrx1*^*−/−*^ 2D2 mice, suggesting the presence of subclinical tissue damage in the CNS.

A recent study showed that neurotoxic glia, known as A1 astrocytes, are induced by microglial inflammatory mediators such as IL-1α, TNFα, and complement component 1q (C1q) and induce the death of neurons and oligodendrocytes [46]. Our study demonstrates that NLRX1 reduces the level of neurotoxic glia. We observed the similar expression pattern of A1 astrocyte genes in the brains of *Nlrx1*^*−/−*^ mice regardless of the presence or absence of a transgenic TCR gene, indicating that the generation of A1 astrocytes is independent of myelin-specific T cells. Consistent with these findings, we found that after LPS/IFNγ treatment, *Nlrx1*^*−/−*^ glia had an increased expression of A1-associated transcripts and a higher production of TNFα compared with WT glia. This resulted in increased cytotoxicity of *Nlrx1*^*−/−*^ glia, as conditioned medium from *Nlrx1*^*−/−*^ cultures induced significantly more cell death in N2A neuroblastoma and MO3.13 oligodendrocyte cell lines. Our results are based on mixed-glial cultures, and the astrocyte intrinsic role of NLRX1 in the CNS inflammation needs further investigation.

Taken together, our study demonstrates the crucial role of the inflammatory status of the innate immune compartment in the development of spEAE. We found that NLRX1 plays a key role in guarding the CNS and preventing the onset of inflammation. On one hand, NLRX1 intrinsically inhibits the autoreactive T-cell response and on the other hand, it protects against the activation of innate immune cells in the CNS (S10 Fig). Several studies have previously demonstrated that microglia respond ahead of T-cell activation and infiltration to the CNS in EAE and MS [41,47–49]. For example, Davalos and colleagues demonstrated that fibrinogen may leak and trigger formation of microglial clusters ahead of myelin loss and paralysis in EAE [47]. In MS, Van der Valk describes preactive lesions, which are characterized by the presence of activated microglial clusters before the appearance of demyelination and leukocyte infiltration in white matter [49]. Consistent with these findings, our study shows that NLRX1 suppresses the generation of inflammatory microglia and neurotoxic astrocytes at the subclinical stage that potentially play a destructive role in the CNS by inducing death in neurons and oligodendrocytes. The nature of the trigger that activates the initial innate response in MS remains unknown.

We previously published that NLRX1 plays a protective role in neuronal cell death [17]. The potential role of NLRX1 in survival of oligodendrocytes and neurons adds another layer of complexity to the NLRX1-dependent pathophysiology of MS and needs further investigation. In humans with MS, NLRX1 may control the level of inflammatory cytokines and keeps the activation status of the peripheral immune system in check. The identification of rare *NLRX1* mutations in MS patients, particularly p.Lys172Asn, p.Glu192Ter, and p.Arg860Trp, supports this hypothesis. However, given their low frequency, further analysis in larger cohorts of MS patients is needed to confirm their role in the onset of disease.

## Materials and methods

### Ethics statement

All the protocols and procedures for the mice studies were approved by Comités d’éthique de la recherche, Université de Sherbrooke (Protocols 280–15 and 335-17B). The animal care and use protocol adhered to Canadian Council on Animal Care (CCAC) regulations/guidelines. The human studies were approved with informed consent by Centre intégré universitaire de santé et de services sociaux de l'Estrie—Centre hospitalier universitaire de Sherbrooke (Project# 2017–1512) and the ethical review board at the University of British Columbia (Project# H08-01669).

### Mice

The mice were maintained under specific pathogen-free conditions in the animal facility of the faculty of medicine at the University of Sherbrooke. *Nlrx1*^*−/−*^ mice (C57BL/6J) were kindly provided by Dr. Jenny P. Y. Ting (Chapel Hill, NC) and the 2D2 TCR transgenic mice were purchased from Jackson Laboratory, Bar Harbor, ME. *Nlrx1*^*−/−*^ mice were crossed with 2D2 mice or *Rag2*^*−/−*^ C57BL/6J mice (kindly provided by Dr. A. Amrani, Sherbrooke, Canada) to generate *Nlrx1*^*−/−*^ 2D2 or *Nlrx1*^*−/−*^*Rag*^*−/−*^ mice, respectively. The 2D2 mice were monitored for the development of spEAE. Animals were euthanized after 4 months of monitoring or after the development of EAE at the peak score of 4. Samples from WT or knockout mice were collected from littermates.

### Immunization and EAE induction

EAE was induced in WT or *Nlrx1*^*−/−*^ female mice as previously described [50]. A mixture of MOG_35−55_ (Genemed Synthesis, San Antonio, TX), CFA (Sigma-Aldrich, St. Louis, MO), and *Mycobacterium tuberculosis* H37 RA (Difco Laboratories, Detroit, MI) was injected subcutaneously into mice, with each mouse receiving a total of 200 μg MOG_35–55_ and 500 μg *Mycobacterium*. PTX (List Biological Laboratories, Campbell, CA) (200 ng) was injected intraperitoneally on days 0 and 2. Mice were euthanized after 3 weeks of immunization, and the tissues were collected.

### Histological analysis

Mice were euthanized, perfused with ice-cold PBS (Wisent, St. Bruno, QC, Canada), and the spinal cords were removed and fixed in 4% formaldehyde for 24 hours. T5-μm sections of lumbar spinal cord were used for hematoxylin–eosin (HE) or immunofluorescence staining of the following markers: CD3 (Abcam, Cambridge, MA, ab5690), GFAP (Cell signaling Technology, Danvers, MA,12389), Iba1 (Wako, Osaka, Japan, 019–19741), MBP (Abcam, Cambridge, MA, ab40390), or IgG (Cell signaling Technology, Danvers, MA, 4408). All slides were scanned using a digital slide scanner NanoZoomer-XR C12000 (Hamamatsu, Hamamatsu City, Japan) and viewed using NDPview2 software (Hamamatsu). The percentage of positive area of the stained markers was quantified after thresholding of images using Fiji (ImageJ) software (NIH). Antibody dilutions were prepared based on the manufacturer’s instruction.

### Flow cytometric analysis of CNS-infiltrating mononuclear cells

CNS tissue was digested with 2.5 mg/mL collagenase D (Roche Diagnostics, Indianapolis, IN, 11088866001) and 1 mg/mL DNase I (Sigma-Aldrich, St. Louis, MO, 11284932001) and filtered through a 70-μm nylon sieve as described previously [12]. Mononuclear cells were isolated by percoll (Sigma-Aldrich, St. Louis, MO) centrifugation. The samples were centrifuged at 1,000*g* for 15 minutes without break, washed, stained for surface markers, and analyzed by flow cytometry. Ten thousand events were acquired in FSC/SSC gate on single cells. Sample acquisition was performed with Beckman Coulter CytoFlex and data were analyzed using CytExpert 2 software (Beckman Coulter). Myeloid markers included anti-CD45-FITC (11-0451-82), anti-CD11b-PE (12-0112-81), and anti-MHCII-PE-Cy5 (15-5321-81) and lymphoid markers included anti-CD4-FITC (11-0042-82), anti-Vβ11-APC (17-5827-82), and anti-CD19-PE (12-0193-81) antibodies. All antibodies were purchased from ThermoFisher Scientific, San Diego, CA.

### T-cell activation and differentiation in vitro

CD4^+^ T cells were purified from the single cell suspension prepared from lymph nodes and spleens using MagniSort CD4 T cell Enrichment Kit (ThermoFisher, San Diego, CA, 8804-6821-74) and activated with MOG-pulsed splenocytes for indicated times. T-cell proliferation was quantified using ^3^H-thymidine incorporation assay and Ki67 intranuclear staining following fixation and permeabilization using Foxp3/Transcription Factor staining kit (eBioscience, San Diego, CA, 00-5523-00). Intracellular staining of cytokines was performed as previously described [12]. Sample acquisition was performed with Beckman Coulter CytoFlex and data were analyzed using CytExpert 2 software (Beckman Coulter).

### Quantitative RT-PCR

RNA was extracted from cells using TRIzol (Life Technologies, Burlington, ON) and cDNA was synthesized as previously described [50]. Primer sequences are presented in S4 Table. The relative expression was calculated using the ΔΔC_T_ method [51].

### ELISA

The level of IFNγ or TNFα in the cell culture supernatants were measured using ELISA kits as described by the manufacturer (PeproTech, Rocky Hill, NJ, 900-T98 and 900-T54 respectively). Blood was collected via cardiac puncture and the serum level of anti-MOG IgG was quantified using ELISA assay as described by Mantegazza and colleagues. [52]. Briefly, a Nunc MaxiSorp 96-well ELISA plate (ThermoFisher, Ottawa, Canada) was coated overnight at 4°C with 100 μL/well of MOG_35−55_ antigen (10 mg/mL) diluted in PBS. After washing with PBS plus 0.05% Tween-20, the plates were blocked with PBS, 1% bovine serum albumin (Sigma Alderich, St. Louis, MO, A8806), for 2 hours at room temperature. The plates were then washed three times, and diluted serum (1:10 in wash buffer plus 1% BSA) was added to each well for 1 hour at room temperature. After washing, the wells were incubated with 100 μL/well of horseradish peroxidase (HRP)-conjugated anti-IgG secondary antibody for 1 hour at room temperature. After washing, TMB substrate was added to each well and the color change was stopped using 0.5 molar sulfuric acid. The optical density (OD) was measured at a wavelength of 450 nm.

### Western blotting

Tissues were homogenized in the lysis buffer plus proteinase and phosphatase inhibitor (Cell Signaling Technology, Danvers, MA). Proteins were measured and separated on SDS-polyacrylamide gels (12%) and transferred to nitrocellulose membrane. After blocking with 5% nonfat milk in TBS/0.1% Tween 20 (TBST) for 1 hour at room temperature, membranes were incubated overnight at 4 °C in the Anti-HMGB1 antibody (Cell Signaling Technology, Danvers, MA, 3935) diluted in TBST. The membranes were washed three times with TBST and incubated in HRP-conjugated goat anti-rabbit antibody (Cell Signaling Technology, Danvers, MA) diluted 1/1,000 in TBST for 1 hour at room temperature. The immunoblots were developed with Lumigen ECL ultra reagent, imaged with ChemiDoc (Bio-Rad), and analyzed using Image Lab software.

### Adoptive transfer

CD4^+^ T cells were purified from spleen and lymph nodes of 2D2 mice and activated with MOG_35–55_ for 48 hours. Then, the cells were harvested, washed, and resuspended in 500 μL of sterile PBS for a total of 3 × 10^6^ CD4^+^ T cells. T cells were transferred intraperitoneally into *Rag*^*−/−*^ or *Nlrx1*^*−/−*^*Rag*^*−/−*^ mice, followed by MOG-CFA immunization as described in the immunization section. Only a single PTX injection (500 ng) was performed intraperitoneally for each animal.

### The preparation of glia-conditioned medium

Brains were extracted and meninges removed from 1-day-old pups. Tissue was chopped and passed through a 70-μm filter. The cells were cultured in DMEM/F12 medium with 10% FBS (Invitrogen, Burlington, Canada) supplemented by 1% penicillin-streptomycin solution, 1% L-glutamine solution, 0.9% sodium pyruvate solution, 0.9% MEM amino acid solution, and 0.9% amphotericin B solution (all from Wisent, St. Bruno, Canada). The medium of the mixed glial culture was changed every 2 to 3 days. Primary glial cells were ready for experiments after 3 weeks. Glial cells were treated with LPS (100 ng/mL) and/or IFNγ (10 ng/mL) for 24 hours, and the conditioned media were collected and stored at −80°C.

### Cell cytotoxicity assay

Human Glial (Oligodendrocytic) Hybrid Cell Line MO3.13 was kindly provided by Dr. Nathalie Arbour, CRCHUM, Montreal, Canada, and N2A neuroblastoma cell line was purchased from American Type Culture Collection. N2A or MO3.13 cells (1 × 10^4^) were grown in 96-well plates in DMEM/F12 medium plus 10% FBS for 24 hours. Thereafter, the medium was replaced with astrocyte-conditioned medium and the incubation was continued for an additional 24 hours. The effect of glia-conditioned medium on the viability of target cell lines, N2A and MO3.13, was determined by MTT assay, as described previously [53]. Cytotoxicity percentage was calculated by the following formula: 100 − (absorbance of treated cells/absorbance of corresponding control × 100). Flow cytometric analysis of cell death was done with Annexin V Apoptosis Detection Kit per the manufacturer's instructions (eBioscience, Diego, CA, 88-8007-72).

### MS subjects and NLRX1 expression

Patients diagnosed with relapsing remitting MS (*n* = 18) based on the revised McDonald Diagnostic Criteria were recruited from the Multiple Sclerosis Clinic at the University of Sherbrooke by a board-certified neurologist. On the day of blood sampling, all subjects were afebrile and had no signs and symptoms of infection based on history, physical examination, and responses to a survey. The age-matched control group consisted of 23 healthy volunteers. The group’s demographic and clinical data are shown in S2 Table. PBMCs were separated by ficoll and RNA extraction was done using Trizol. The CD3^+^ T cells and CD14^+^ monocytes were sorted from frozen PBMCs using BD FACSAriaII (70-μm nozzle) at Johns Hopkins Sidney Kimmel Cancer Center Flow Cytometry and Immune Monitoring Core facility. Cells were stained with CD14 FITC and CD3 APCVio770 (Miltenyi). Cell viability was determined using Helix NP Blue (Biolegend). The expression of NLRX1 was quantified using qPCR and normalized to the internal control GAPDH.

### Genetic methods

Biological samples from 2,480 MS patients and 1,024 healthy controls were collected through the longitudinal Canadian Collaborative Project on the Genetic Susceptibility to Multiple Sclerosis (CCPGSMS) [54]. All patients were diagnosed with MS according to Poser criteria prior to 2001 [55], or McDonald criteria thereafter [56]. Cohort demographics have been described elsewhere [57]. Exome sequencing data from 326 MS patients and 123 healthy controls were generated as previously described [58], and variants of interest were genotyped using TaqMan probes. Sanger sequencing was used to confirm non-reference genotype calls and to assess segregation within families as previously described [59].

### Statistical analysis

Statistical analyses were conducted using GraphPad Prism 7 software. For each set of data, “*N*” represents the number of independent samples. Results were expressed as the mean ± standard deviation of at least two independent experiments. The degree of normality was determined using Shapiro-Wilk test and the equality of variance was evaluated with an F test. If the samples followed a normal distribution, the unpaired Student *t* test was applied. In the absence of normality, the nonparametric Mann–Whitney *U* test was used to assess the statistical differences between two independent groups. For comparing more than two independent groups, we used the one-way ANOVA followed by the Tukey multiple comparison test. The significance level was set at *P* ≤ 0.05.

## Supporting information

S1 Table *Nlrx1*^*−/−*^ *2D2* model compared to previous models with CD4 T cells involvement. *Nlrx1*^*−/−*^, nucleotide-binding, leucine-rich repeat containing X1 knockout.
(XLSX)

S2 Table Demographic data of MS patients and healthy individuals. *MS disease modifying drugs including teriflumonide, *n* = 4; dimethyl fumarate, *n* = 3; fingolimod, *n* = 1; natalizumab, *n* = 1; glatiramer acetate, *n* = 1; interferon beta-1a, *n* = 4. MS, multiple sclerosis; RRMS, replacing remitting MS.
(XLSX)

S3 Table NLRX1 mutations identified in MS patients. Genomic coordinates from NCBI Build 37.1 (hg19) and dbSNP refSNP (rs) identifiers from build 150 are provided. Estimated effect on protein function was assessed with CADD phred-scale scores. Sample counts and/or minor allele frequency (MAF) for the Genome Aggregation Database (gnomAD), MS patients, and healthy controls are given. CADD, Combined Annotation Dependent Depletion; dbSNP, Single Nucleotide Polymorphism Database; hg19, human genome version 19; MAF, minor allele frequency; MS, multiple sclerosis; NA, not available; NLRX1, nucleotide-binding, leucine-rich repeat containing X1; refSNP (rs), reference SNP.
(XLSX)

S4 Table The primer sequences used for qPCR experiments. qPCR, quantitative polymerase chain reaction.
(XLSX)

S1 Fig **(A)** The percentage of positive area in spinal cords from healthy 2D2 and *Nlrx1*^*−/−*^2D2 mice stained for GFAP, Iba1, and MBP markers. **(B)** The mRNA levels of IFNα and IFNβ in the brains of WT and *Nlrx1*^*−/−*^ mice in healthy and disease conditions. (**C)** Nuclear localization of NF-κB p65 subunit in the focal lesions of a spEAE spinal cord, pink nuclei shown by white arrows; confocal microscope 63× magnification. All the data are presented in mean ± SD. *P ≤ 0.05, **P ≤ 0.01, ***P ≤ 0.001, determined by one-way ANOVA. Underlying data can be found in S1 Data. GFAP, glial fibrillary acidic protein; Iba1, ionized calcium binding adaptor molecule 1; IFNα, interferon alpha; IFNβ, interferon beta; MBP, myelin basic protein; NF-κB, nuclear factor κB; *Nlrx1*, nucleotide-binding, leucine-rich repeat containing X1; spEAE, spontaneous EAE; WT, wild-type.
(TIF)

S2 Fig Nuclear localization of NF-κB p65 in astrocytes from *Nlrx1*^*−/−*^ 2D2 spEAE mice. Confocal microscope 63× magnification of p65 in GFAP^+^ astrocytes. The white arrows show the representative cells. GFAP, glial fibrillary acidic protein; NF-κB, nuclear factor κB; spEAE, spontaneous EAE.
(TIF)

S3 Fig The status of T-cell activation in healthy and spEAE mice. **(A)** The percentages of myelin-specific Vβ11^+^ T cells in the spleens of 2D2 and *Nlrx1*^*−/−*^ 2D2 mice in the healthy and spEAE status, quantified by flow cytometry. **(B)** The mRNA levels of T-cell activation markers, CD44 and CD25, in the lymph nodes of 2D2 and *Nlrx1*^*−/−*^ 2D2 mice in the healthy and spEAE status, quantified by qPCR. **(C)** The expression of Th1 transcription factor, Tbet, and IL-17 cytokine in the lymph nodes of 2D2 and *Nlrx1*^*−/−*^ 2D2 mice in the healthy and spEAE status, quantified by qPCR. All data are presented as mean ± SD. *P ≤ 0.05, as determined by the two-tailed Student *t* test or one-way ANOVA. Underlying data can be found in S1 Data. IL-17, interleukin 7; *Nlrx1*, nucleotide-binding, leucine-rich repeat containing X1; qPCR, quantitative polymerase chain reaction; spEAE, spontaneous EAE; Tbet, T-Box transcription factor; Th, T helper.
(JPG)

S4 Fig *Nlrx1*^*−/−*^APC activate T cells as efficiently as WT APC. **(A)** The expression of T-cell activation markers (CD69 and CD25) by 2D2 T cells activated with MOG in the presence of WT APC or *Nlrx1*^*−/−*^APC for 24 hours, quantified by flow cytometry (*n* = 4). **(B)** The expression of proliferation marker Ki67 by MOG-activated 2D2 T cells in the presence of MOG-pulsed WT APC or *Nlrx1*^*−/−*^APC for 48 hours (*n* = 4). **(C)** A representative flow cytometry plot showing the peak of proliferating CD4^+^Ki67^+^ T cells activated by MOG-pulsed WT splenocytes (blue line) or *Nlrx1*^*−/−*^splenocytes (red line) for 24 hours. **(D)** The differentiation of T cells to inflammatory T-cell subtypes (Th1 and Th17) by MOG-activated 2D2 T cells in the presence of WT APC or *Nlrx1*^*−/−*^APC and polarizing cytokines for 72 hours, quantified by flow cytometry (*n* = 4). All the data are presented as mean ± SD. Underlying data can be found in S1 Data. APC, antigen presenting cell; MOG, myelin oligodendrocyte glycoprotein; *Nlrx1*, nucleotide-binding, leucine-rich repeat containing X1; WT, wild-type.
(TIF)

S5 Fig NLRX1 inhibits T-cell activation, proliferation, and differentiation to inflammatory subsets. **(A)** The expression of early activation marker, CD69, in *Nlrx1*^*−/−*^2D2 or 2D2 T cells after a 24-hour activation with MOG (*n* = 5). **(B)** The kinetics of CD25 (IL-2R) expression on *Nlrx1*^*−/−*^ or 2D2 T cells after 24-, 48-, and 72-hour activations with MOG (*n* = 5). **(C)** The proliferation of *Nlrx1*^*−/−*^ 2D2 compared with 2D2 T cells after a 48-hour activation with MOG-pulsed splenocytes. **(D)** The proliferation of *Nlrx1*^*−/−*^2D2 T cells compared with 2D2 T cells after a 24-hour activation with MOG-pulsed splenocytes, quantified using Ki67 staining and flow cytometry. **(E)** A representative flow cytometry plot showing the higher peak of proliferating CD4^+^Ki67^+^ *Nlrx1*^*−/−*^ T cells (red line) compared with CD4^+^Ki67^+^ WT T cells (blue line) after a 24-hour activation. **(F)** The production of IFNγ by activated *Nlrx1*^*−/−*^2D2 T cells compared with 2D2 T cells quantified by ELISA. **(G)** Flow cytometric analysis of IFNγ^+^CD4^+^ T cells in *Nlrx1*^*−/−*^2D2 T cells or 2D2 T cells after a 48-hour activation with MOG-pulsed splenocytes (*n* = 6). (**H)** Flow cytometric quantification of *Nlrx1*^*−/−*^2D2 or 2D2 T cells differentiation to Th1 (IFNγ^+^CD4^+^ T cells) or Th17 (RORγt^+^CD4^+^ T cells) activated with MOG-pulsed splenocytes for 72 hours in the presence of Th1 or Th17 polarizing cytokines (*n* = 4). All the data are presented in mean ± SD. *P ≤ 0.05, determined by the two-tailed Student *t* test. Underlying data can be found in S1 Data. IFNγ, interferon gamma; IL-2R, interleukin 2 receptor; MOG, myelin oligodendrocyte glycoprotein; NLRX1, nucleotide-binding, leucine-rich repeat containing X1; Th, T helper; WT, wild-type.
(TIF)

S6 Fig Increased levels of IgG and frequency of B cells in the spinal cords of *Nlrx1*^*−/−*^2D2 spEAE mice. **(A)** Representative western blot of IgG in the spinal cords of *Nlrx1*^*−/−*^*2D2* spEAE mice and healthy mice. **(B)** Quantitative analysis of IgG/β-tubulin ratio in healthy and *Nlrx1*^*−/−*^*2D2* spEAE spinal cords (*n* = 6 mice per group). **(C)** Representative images of immunofluorescence staining for IgG leakage into the spinal cords of *Nlrx1*^*−/−*^2D2 spEAE mice and healthy spinal cord sections, magnification 40×. **(D)** The percentage of CD19^+^ B cells in the spinal cords and brains of *Nlrx1*^*−/−*^*2D2* spEAE mice compared with healthy mice (*n* = 8 mice per group). **(E)** Flow cytometry analysis of CD45^+^CD19^+^ B cells in the spinal cord of healthy and spEAE mice. **(F)** Serum levels of anti-MOG IgG in *Nlrx1*^*−/−*^*2D2* spEAE and healthy mice (*n* = 4 mice per group), measured by ELISA; mean absorbance at OD 450 nm is shown. All data are presented as mean ± SD. *P ≤ 0.05, as determined by the two-tailed Student *t* test. Underlying data can be found in S1 Data. IgG, immunoglobulin G; MOG, myelin oligodendrocyte glycoprotein; *Nlrx1*, nucleotide-binding, leucine-rich repeat containing X1; OD, optical density; spEAE, spontaneous EAE.
(TIF)

S7 Fig **(A)** Representative flow cytometry plots showing the CD45 low and high gating strategies based on unstained and CD45-stained splenocytes (negative and positive controls, respectively). **(B)** Flow cytometric analysis of CD45^high^ cells in the brain of MOG-CFA/pertussis immunized *Rag*^*−/−*^ and *Nlrx1*^*−/−*^*Rag*^*−/−*^ mice. **(C)** The infiltration of CD45^high^ leukocytes to the spinal cords of *Nlrx1*^*−/−*^*Rag*^*−/−*^ mice compared with *Rag*^*−/−*^ mice 14 days after immunization with MOG-CFA emulsion plus PTX, quantified by flow cytometry as shown in representative plots, *P ≤ 0.05, as determined by ANOVA test. **(D)** The percentage of activated CD11b^+^MHCII^+^ microglia/macrophages in CD45^+^ cells, quantified by flow cytometry. **(E)** The mRNA levels of T cell–associated markers in the spleens of *Rag*^*−/−*^ and *Nlrx1*^*−/−*^*Rag*^*−/−*^ mice after 3 weeks of adoptive transfer experiment. Underlying data can be found in S1 Data. CFA, Complete Freund's Adjuvant; MOG, myelin oligodendrocyte glycoprotein; *Nlrx1*, nucleotide-binding, leucine-rich repeat containing X1; PTX, pertussis toxin; *Rag*, recombination-activating gene.
(TIF)

S8 Fig NLRX1 expression in T cells and monocytes in PBMC from MS patients. **(A)** The purity of CD14^+^ cells and CD3^+^ cells isolated from PBMC using a fluorescence activated cell sorter. **(B)** The mRNA levels of NLRX1 were quantified in CD14^+^ cells relative to CD3^+^ cells from each individual using qPCR (*n* = 3). Underlying data can be found in S1 Data. MS, multiple sclerosis; NLRX1, nucleotide-binding, leucine-rich repeat containing X1; PBMC, peripheral blood mononuclear cell; qPCR, quantitative polymerase chain reaction.
(TIF)

S9 Fig Protein conservation in orthologs and human paralogs. Organism and RefSeq accession numbers are provided for orthologs and gene name and RefSeq accession numbers for human paralogs, which were obtained from Ensembl release 91. Evolutionarily conserved positions for the NLRX1 K172N mutation is highlighted in black.
(TIF)

S10 Fig The hypothetical mechanism of NLRX1 function in the early stages of CNS inflammation and the onset of EAE. Failure to maintain proper inflammatory balance in *Nlrx1*^*−/−*^ microglia produces a milieu that promotes toxic A1 astrocyte phenotype (A1). This results in damage to neurons and oligodendrocytes and creates a T-cell chemoattractant gradient. Upon activation and differentiation in secondary lymphoid organs, Th1-biased T cells migrate to the CNS and induce autoimmune attack. Subclinical stage, phase 1: from previous studies, we know that unknown factors trigger the inflammatory pathways in microglia [60]. This may lead to the production of inflammatory cytokines such as TNFα and IL-1β that promote generation of neurotoxic A1 astrocytes. As a result, a limited number of oligodendrocytes die, and myelin antigen is drained to the deep cervical lymph nodes. At the same time, A1 astrocytes increase expression of T-cell chemokines such as CCL20. Subclinical stage, phase 2: in the lymph nodes, autoreactive T cells proliferate and differentiate into encephalitogenic T-cell subsets (Th1, Th17). Clinical stage, phase 3: clonal expansion of autoreactive encephalitogenic T cells, activation of myeloid cells such as monocytes and macrophages, and production of inflammatory mediators leads to their excavation into the CNS. Clinical stage, phase 4: activated T cells and monocyte/macrophages infiltrate into the CNS, interact with hyperactivated glial cells and boost the inflammation, resulting in reactive gliosis, progressive inflammatory demyelination, neurodegeneration, and eventually the appearance of neurological symptoms. NLRX1 has a broad range of regulatory activity, preventing the onset of clinical signs at the levels of the CNS and periphery. Whether *Nlrx1*^*−/−*^ astrocytes themselves are prone to differentiation to the neurotoxic phenotype or *Nlrx1*^*−/−*^ oligodendrocytes are intrinsically susceptible to cell death is still unknown. The sequence of events from a preclinical to a clinical stage warrants further investigation. CCL20, C-C motif chemokine ligand 20; CNS, central nervous system; EAE, experimental autoimmune encephalomyelitis; IL-1β, interleukin 1 beta; NLRX1, nucleotide-binding, leucine-rich repeat containing X1; Th, T helper; TNFα, tumor necrosis factor alpha.
(TIF)

S1 Data Data underlying Figs 1–6, S1 Fig and S3–S8 Figs. (XLSX)

## Abbreviations

APC: antigen presenting cell
BBB: blood-brain barrier
CADD: Combined Annotation Dependent Depletion
CCAC: Canadian Council on Animal Care
CCL20: C-C motif chemokine ligand 20
CCPGSMS: Canadian Collaborative Project on the Genetic Susceptibility to Multiple Sclerosis
Ccr5: C-C chemokine receptor type 5
CFA: Complete Freund's Adjuvant
CNS: central nervous system
C1q: complement component 1q
EAE: experimental autoimmune encephalomyelitis
GFAP: glial fibrillary acidic protein
HE: hematoxylin–eosin
HMGB1: high mobility group box 1
HRP: horseradish peroxidase
Iba1: ionized calcium binding adaptor molecule 1
IFN: interferon
IgG: immunoglobulin G
IL-1: interleukin 1
iNOS: inducible nitric oxide synthase
LPS: lipopolysaccharide
MBP: myelin basic protein
MOG: myelin oligodendrocyte glycoprotein
MS: multiple sclerosis
NF-κB: nuclear factor κB
NLR: nucleotide-binding oligomerization domain, leucine-rich repeat containing protein
NLRP: nucleotide-binding oligomerization domain, leucine rich repeat and pyrin domain containing
NLRX1: nucleotide-binding, leucine-rich repeat containing X1
NOS2: nitric oxide synthase
OD: optical density
PBMC: peripheral blood mononuclear cell
PRR: pattern-recognition receptor
PTX: pertussis toxin
qPCR: quantitative polymerase chain reaction
Rag: recombination-activating gene
RRMS: relapsing-remitting MS
SJL: Swiss Jim Lambert
spEAE: spontaneous EAE
Tbet: T-Box transcription factor
TBST: TBS/0.1% Tween 20
TCR: T-cell receptor
Th: T helper
TLR: toll-like receptor
Tnfα: tumor necrosis factor alpha
Treg: regulatory T
WT: wild-type
