# Supplementary figures and images for: NLRX1 inhibits the early stages of CNS inflammation and prevents the onset of spontaneous autoimmunity
Source: PLoS Biol. 2019 Sep 16;17(9):e3000451. doi: 10.1371/journal.pbio.3000451 (PMC6762215; doi:10.1371/journal.pbio.3000451)

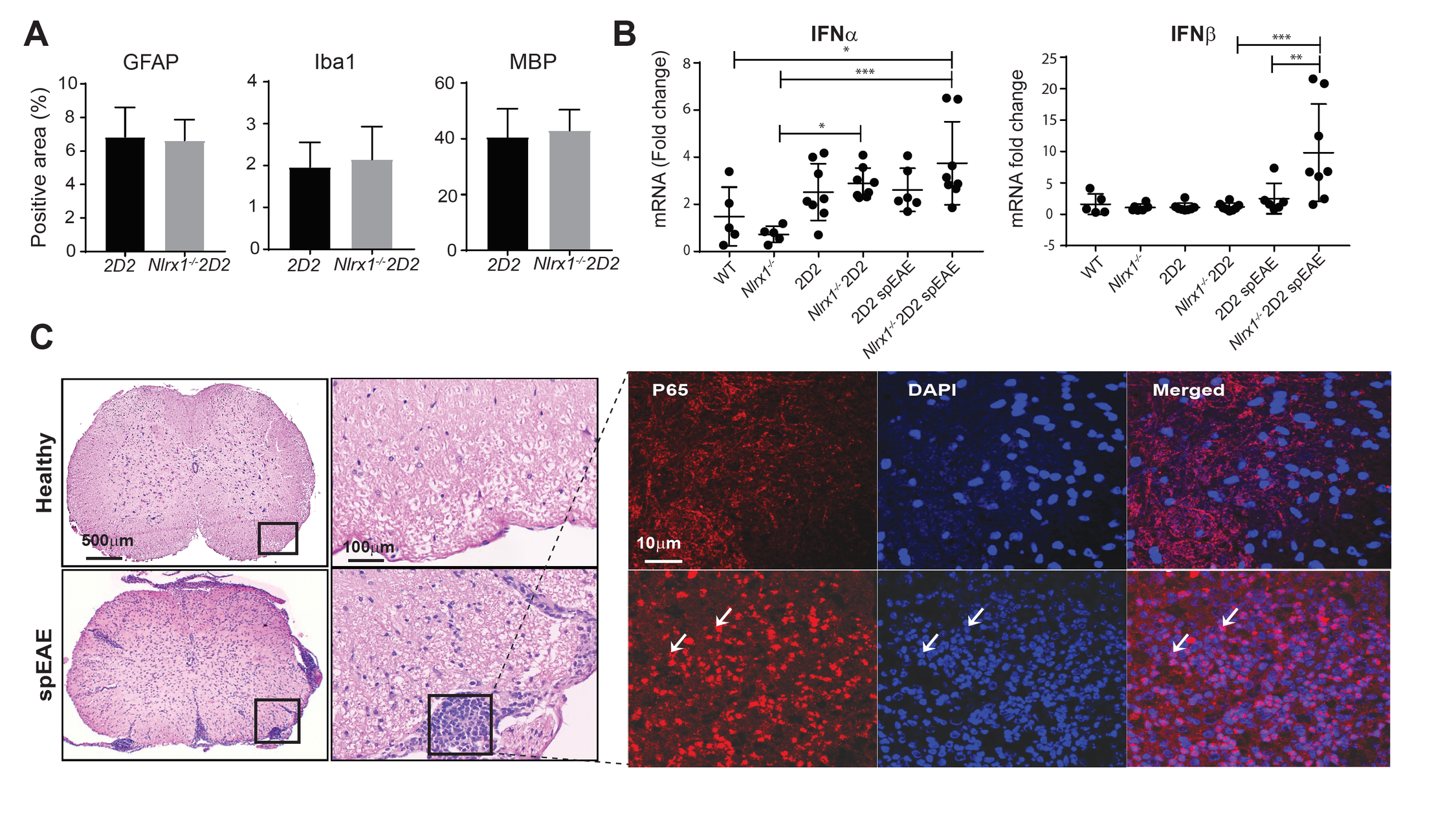

Supplement: S1 Fig — (A) The percentage of positive area in spinal cords from healthy 2D2 and Nlrx1−/−2D2 mice stained for GFAP, Iba1, and MBP markers. (B) The mRNA levels of IFNα and IFNβ in the brains of WT and Nlrx1−/− mice in healthy and disease conditions. (C) Nuclear localization of NF-κB p65 subunit in the focal lesions of a spEAE spinal cord, pink nuclei shown by white arrows; confocal microscope 63× magnification. All the data are presented in mean ± SD. *P ≤ 0.05, **P ≤ 0.01, ***P ≤ 0.001, determined by one-way ANOVA. Underlying data can be found in S1 Data. GFAP, glial fibrillary acidic protein; Iba1, ionized calcium binding adaptor molecule 1; IFNα, interferon alpha; IFNβ, interferon beta; MBP, myelin basic protein; NF-κB, nuclear factor κB; Nlrx1, nucleotide-binding, leucine-rich repeat containing X1; spEAE, spontaneous EAE; WT, wild-type. (TIF) [file pbio.3000451.s005.tif]

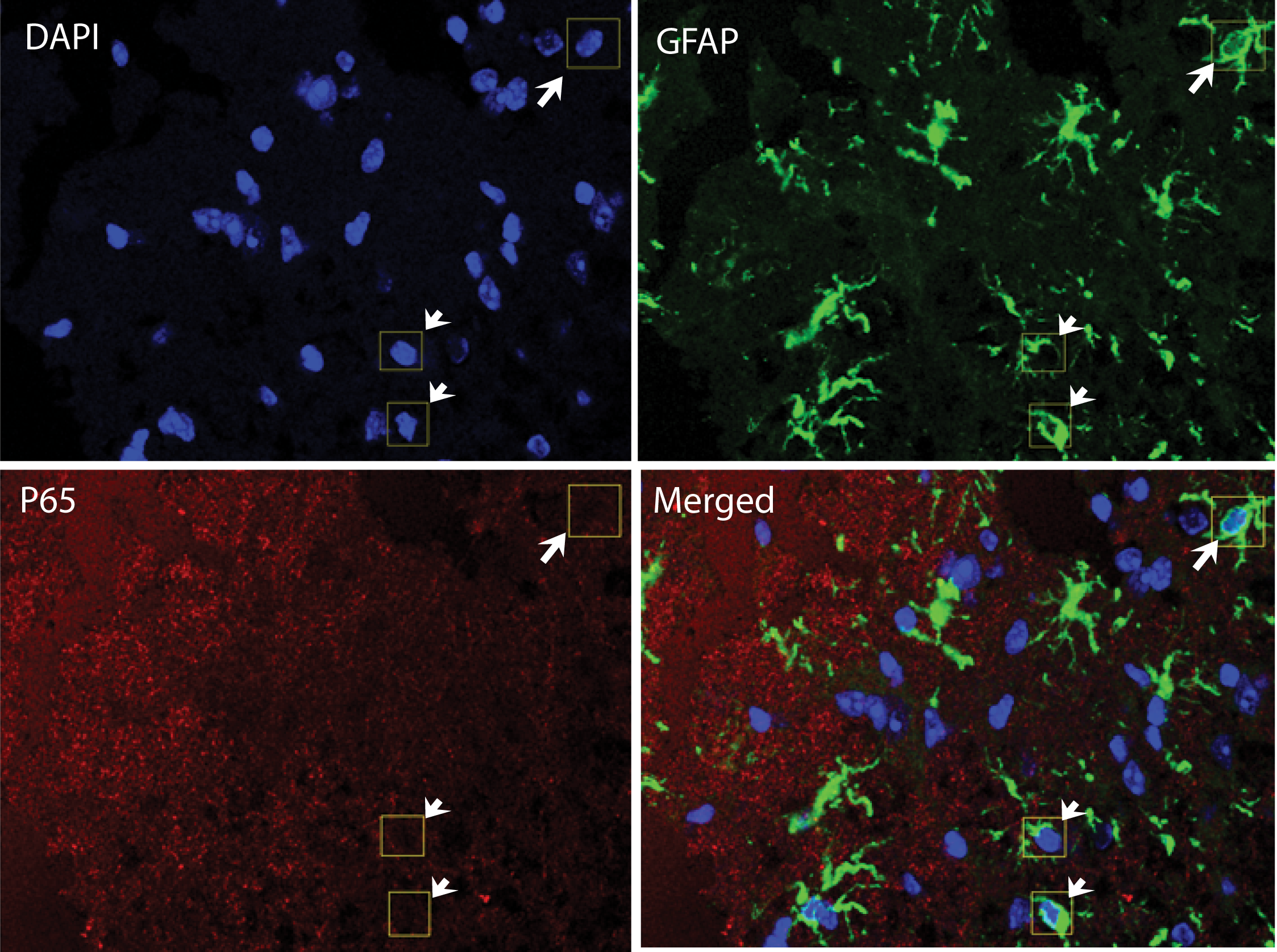

Supplement: S2 Fig — Confocal microscope 63× magnification of p65 in GFAP+ astrocytes. The white arrows show the representative cells. GFAP, glial fibrillary acidic protein; NF-κB, nuclear factor κB; spEAE, spontaneous EAE. (TIF) [file pbio.3000451.s006.tif]

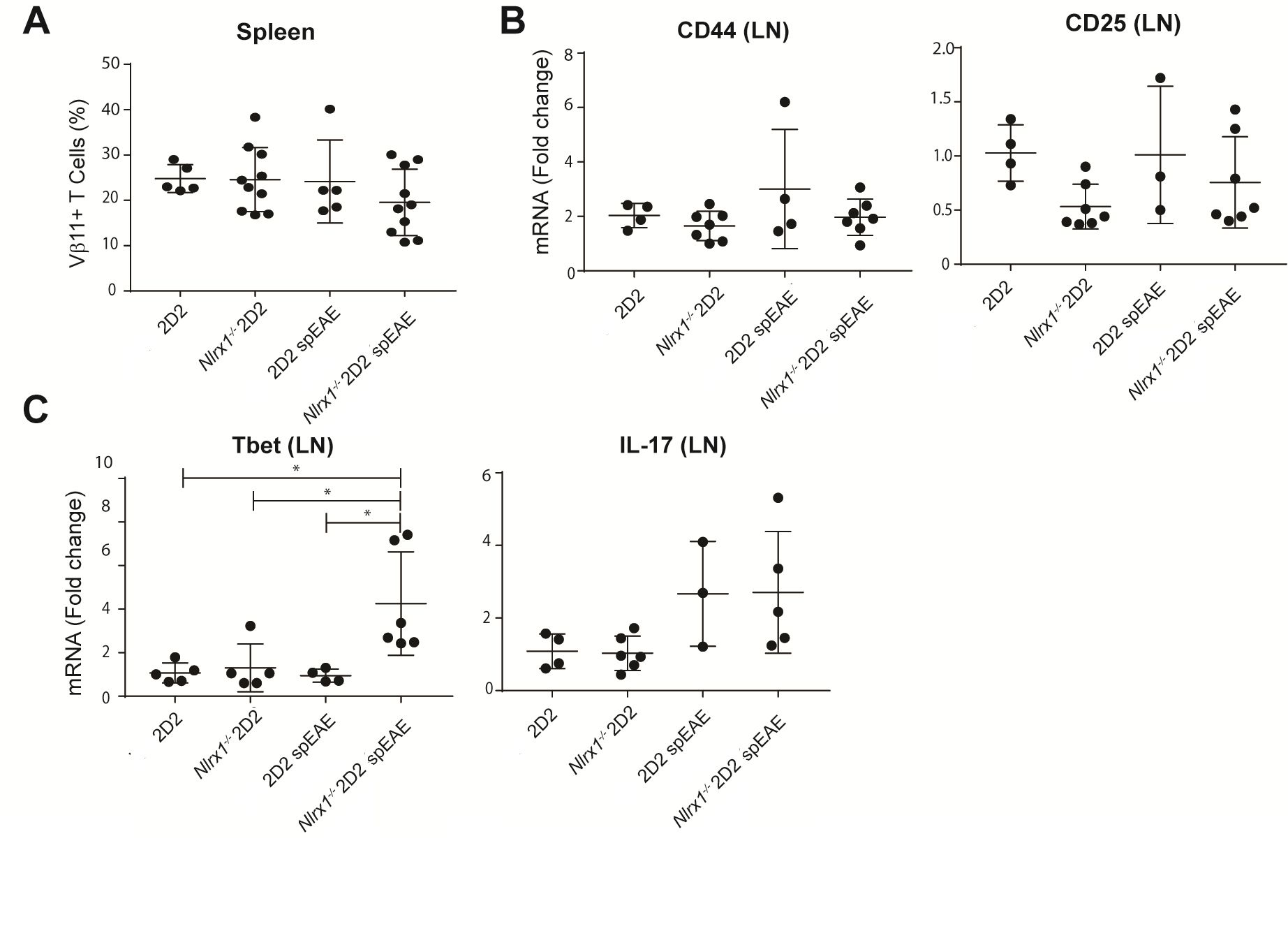

Supplement: S3 Fig — (A) The percentages of myelin-specific Vβ11+ T cells in the spleens of 2D2 and Nlrx1−/− 2D2 mice in the healthy and spEAE status, quantified by flow cytometry. (B) The mRNA levels of T-cell activation markers, CD44 and CD25, in the lymph nodes of 2D2 and Nlrx1−/− 2D2 mice in the healthy and spEAE status, quantified by qPCR. (C) The expression of Th1 transcription factor, Tbet, and IL-17 cytokine in the lymph nodes of 2D2 and Nlrx1−/− 2D2 mice in the healthy and spEAE status, quantified by qPCR. All data are presented as mean ± SD. *P ≤ 0.05, as determined by the two-tailed Student t test or one-way ANOVA. Underlying data can be found in S1 Data. IL-17, interleukin 7; Nlrx1, nucleotide-binding, leucine-rich repeat containing X1; qPCR, quantitative polymerase chain reaction; spEAE, spontaneous EAE; Tbet, T-Box transcription factor; Th, T helper. (JPG) [file pbio.3000451.s007.jpg]

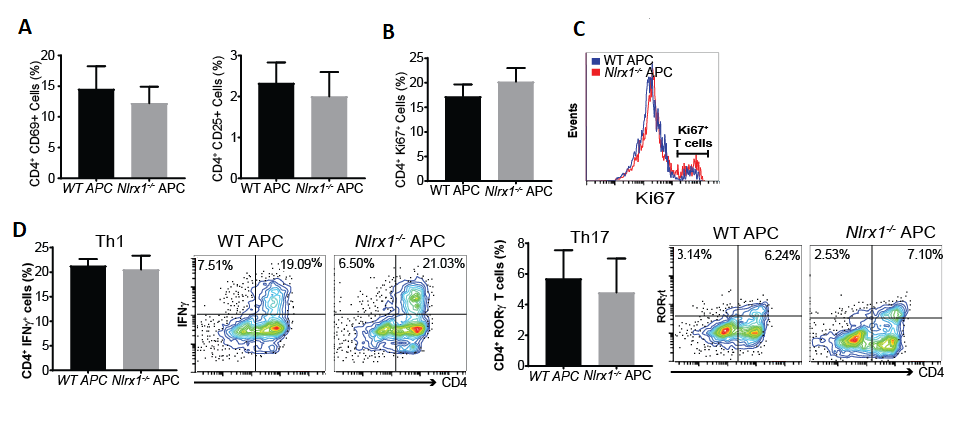

Supplement: S4 Fig — (A) The expression of T-cell activation markers (CD69 and CD25) by 2D2 T cells activated with MOG in the presence of WT APC or Nlrx1−/−APC for 24 hours, quantified by flow cytometry (n = 4). (B) The expression of proliferation marker Ki67 by MOG-activated 2D2 T cells in the presence of MOG-pulsed WT APC or Nlrx1−/−APC for 48 hours (n = 4). (C) A representative flow cytometry plot showing the peak of proliferating CD4+Ki67+ T cells activated by MOG-pulsed WT splenocytes (blue line) or Nlrx1−/−splenocytes (red line) for 24 hours. (D) The differentiation of T cells to inflammatory T-cell subtypes (Th1 and Th17) by MOG-activated 2D2 T cells in the presence of WT APC or Nlrx1−/−APC and polarizing cytokines for 72 hours, quantified by flow cytometry (n = 4). All the data are presented as mean ± SD. Underlying data can be found in S1 Data. APC, antigen presenting cell; MOG, myelin oligodendrocyte glycoprotein; Nlrx1, nucleotide-binding, leucine-rich repeat containing X1; WT, wild-type. (TIF) [file pbio.3000451.s008.tif]

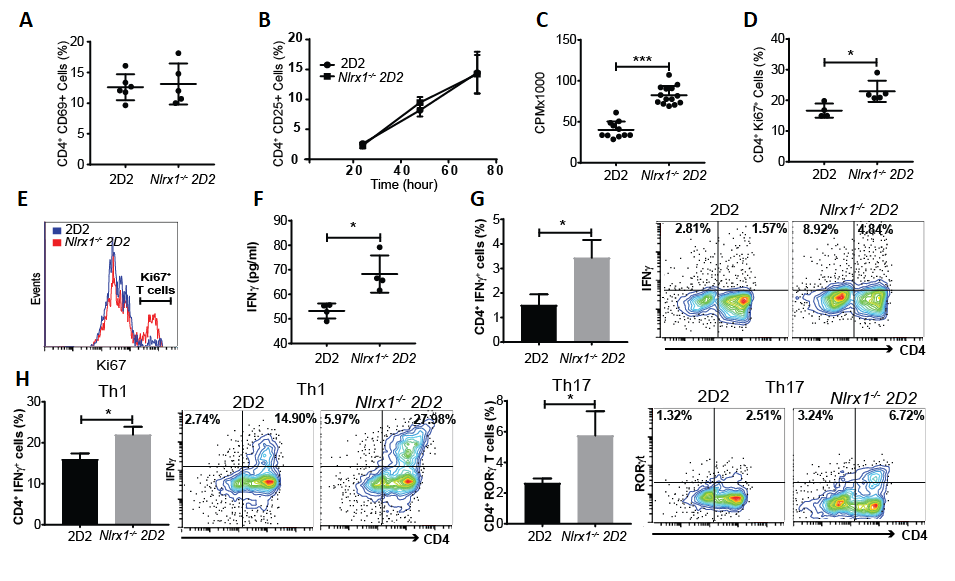

Supplement: S5 Fig — (A) The expression of early activation marker, CD69, in Nlrx1−/−2D2 or 2D2 T cells after a 24-hour activation with MOG (n = 5). (B) The kinetics of CD25 (IL-2R) expression on Nlrx1−/− or 2D2 T cells after 24-, 48-, and 72-hour activations with MOG (n = 5). (C) The proliferation of Nlrx1−/− 2D2 compared with 2D2 T cells after a 48-hour activation with MOG-pulsed splenocytes. (D) The proliferation of Nlrx1−/−2D2 T cells compared with 2D2 T cells after a 24-hour activation with MOG-pulsed splenocytes, quantified using Ki67 staining and flow cytometry. (E) A representative flow cytometry plot showing the higher peak of proliferating CD4+Ki67+ Nlrx1−/− T cells (red line) compared with CD4+Ki67+ WT T cells (blue line) after a 24-hour activation. (F) The production of IFNγ by activated Nlrx1−/−2D2 T cells compared with 2D2 T cells quantified by ELISA. (G) Flow cytometric analysis of IFNγ+CD4+ T cells in Nlrx1−/−2D2 T cells or 2D2 T cells after a 48-hour activation with MOG-pulsed splenocytes (n = 6). (H) Flow cytometric quantification of Nlrx1−/−2D2 or 2D2 T cells differentiation to Th1 (IFNγ+CD4+ T cells) or Th17 (RORγt+CD4+ T cells) activated with MOG-pulsed splenocytes for 72 hours in the presence of Th1 or Th17 polarizing cytokines (n = 4). All the data are presented in mean ± SD. *P ≤ 0.05, determined by the two-tailed Student t test. Underlying data can be found in S1 Data. IFNγ, interferon gamma; IL-2R, interleukin 2 receptor; MOG, myelin oligodendrocyte glycoprotein; NLRX1, nucleotide-binding, leucine-rich repeat containing X1; Th, T helper; WT, wild-type. (TIF) [file pbio.3000451.s009.tif]

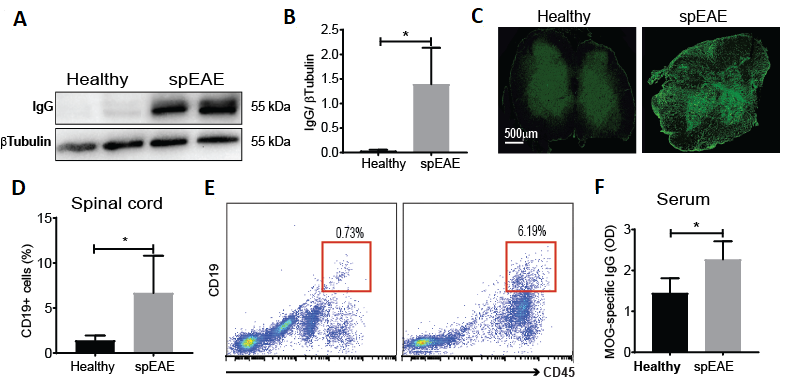

Supplement: S6 Fig — (A) Representative western blot of IgG in the spinal cords of Nlrx1−/−2D2 spEAE mice and healthy mice. (B) Quantitative analysis of IgG/β-tubulin ratio in healthy and Nlrx1−/−2D2 spEAE spinal cords (n = 6 mice per group). (C) Representative images of immunofluorescence staining for IgG leakage into the spinal cords of Nlrx1−/−2D2 spEAE mice and healthy spinal cord sections, magnification 40×. (D) The percentage of CD19+ B cells in the spinal cords and brains of Nlrx1−/−2D2 spEAE mice compared with healthy mice (n = 8 mice per group). (E) Flow cytometry analysis of CD45+CD19+ B cells in the spinal cord of healthy and spEAE mice. (F) Serum levels of anti-MOG IgG in Nlrx1−/−2D2 spEAE and healthy mice (n = 4 mice per group), measured by ELISA; mean absorbance at OD 450 nm is shown. All data are presented as mean ± SD. *P ≤ 0.05, as determined by the two-tailed Student t test. Underlying data can be found in S1 Data. IgG, immunoglobulin G; MOG, myelin oligodendrocyte glycoprotein; Nlrx1, nucleotide-binding, leucine-rich repeat containing X1; OD, optical density; spEAE, spontaneous EAE. (TIF) [file pbio.3000451.s010.tif]

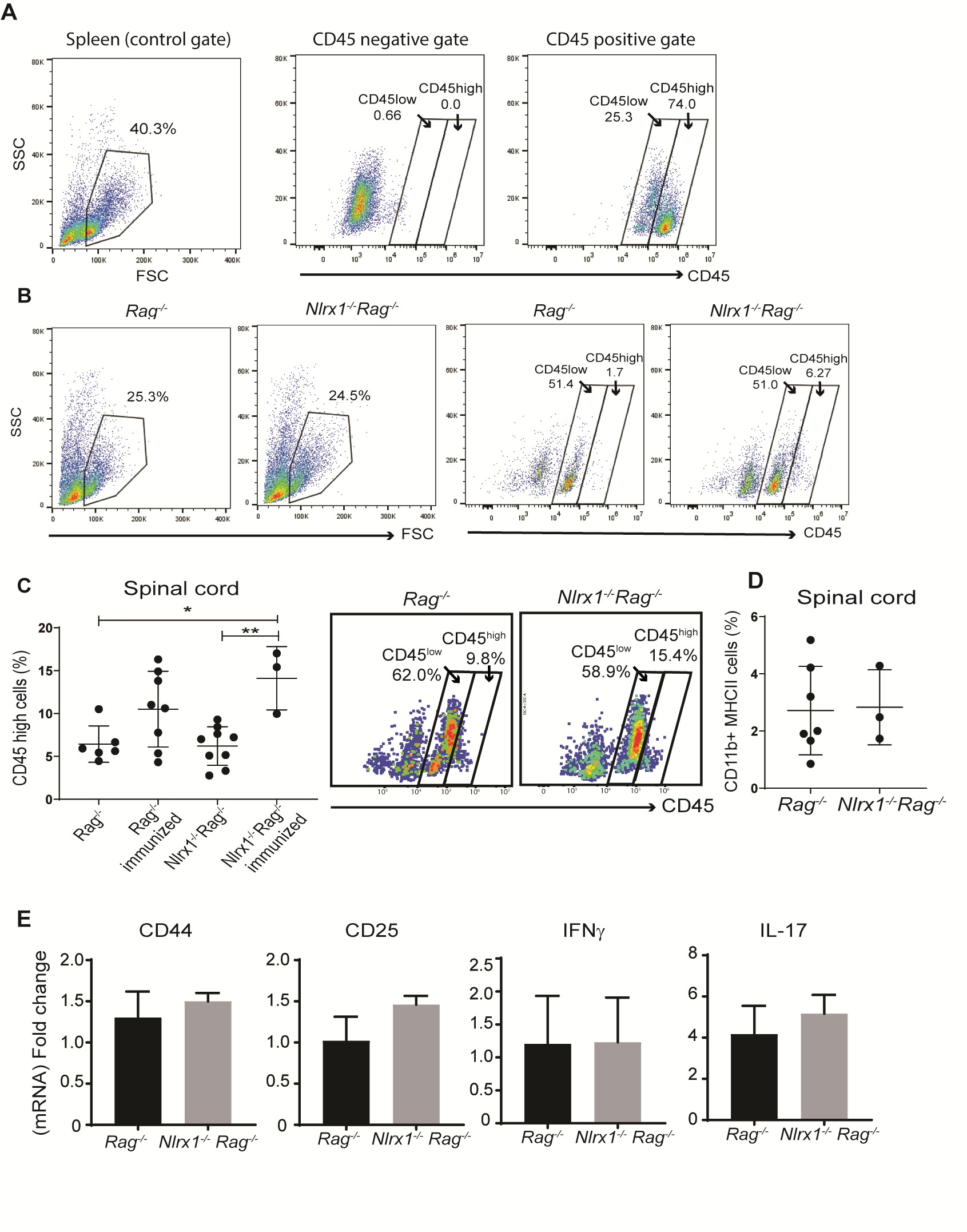

Supplement: S7 Fig — (A) Representative flow cytometry plots showing the CD45 low and high gating strategies based on unstained and CD45-stained splenocytes (negative and positive controls, respectively). (B) Flow cytometric analysis of CD45high cells in the brain of MOG-CFA/pertussis immunized Rag−/− and Nlrx1−/−Rag−/− mice. (C) The infiltration of CD45high leukocytes to the spinal cords of Nlrx1−/−Rag−/− mice compared with Rag−/− mice 14 days after immunization with MOG-CFA emulsion plus PTX, quantified by flow cytometry as shown in representative plots, *P ≤ 0.05, as determined by ANOVA test. (D) The percentage of activated CD11b+MHCII+ microglia/macrophages in CD45+ cells, quantified by flow cytometry. (E) The mRNA levels of T cell–associated markers in the spleens of Rag−/− and Nlrx1−/−Rag−/− mice after 3 weeks of adoptive transfer experiment. Underlying data can be found in S1 Data. CFA, Complete Freund's Adjuvant; MOG, myelin oligodendrocyte glycoprotein; Nlrx1, nucleotide-binding, leucine-rich repeat containing X1; PTX, pertussis toxin; Rag, recombination-activating gene. (TIF) [file pbio.3000451.s011.tif]

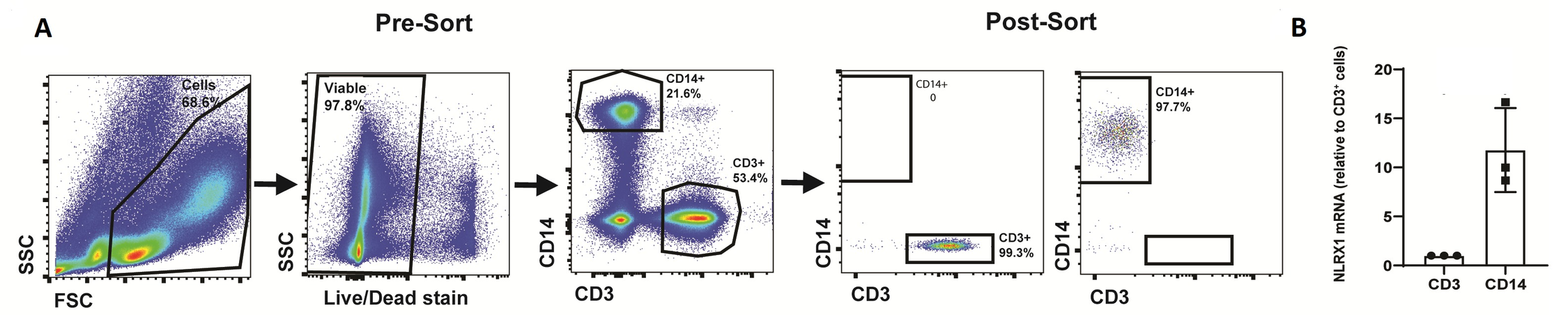

Supplement: S8 Fig — (A) The purity of CD14+ cells and CD3+ cells isolated from PBMC using a fluorescence activated cell sorter. (B) The mRNA levels of NLRX1 were quantified in CD14+ cells relative to CD3+ cells from each individual using qPCR (n = 3). Underlying data can be found in S1 Data. MS, multiple sclerosis; NLRX1, nucleotide-binding, leucine-rich repeat containing X1; PBMC, peripheral blood mononuclear cell; qPCR, quantitative polymerase chain reaction. (TIF) [file pbio.3000451.s012.tif]

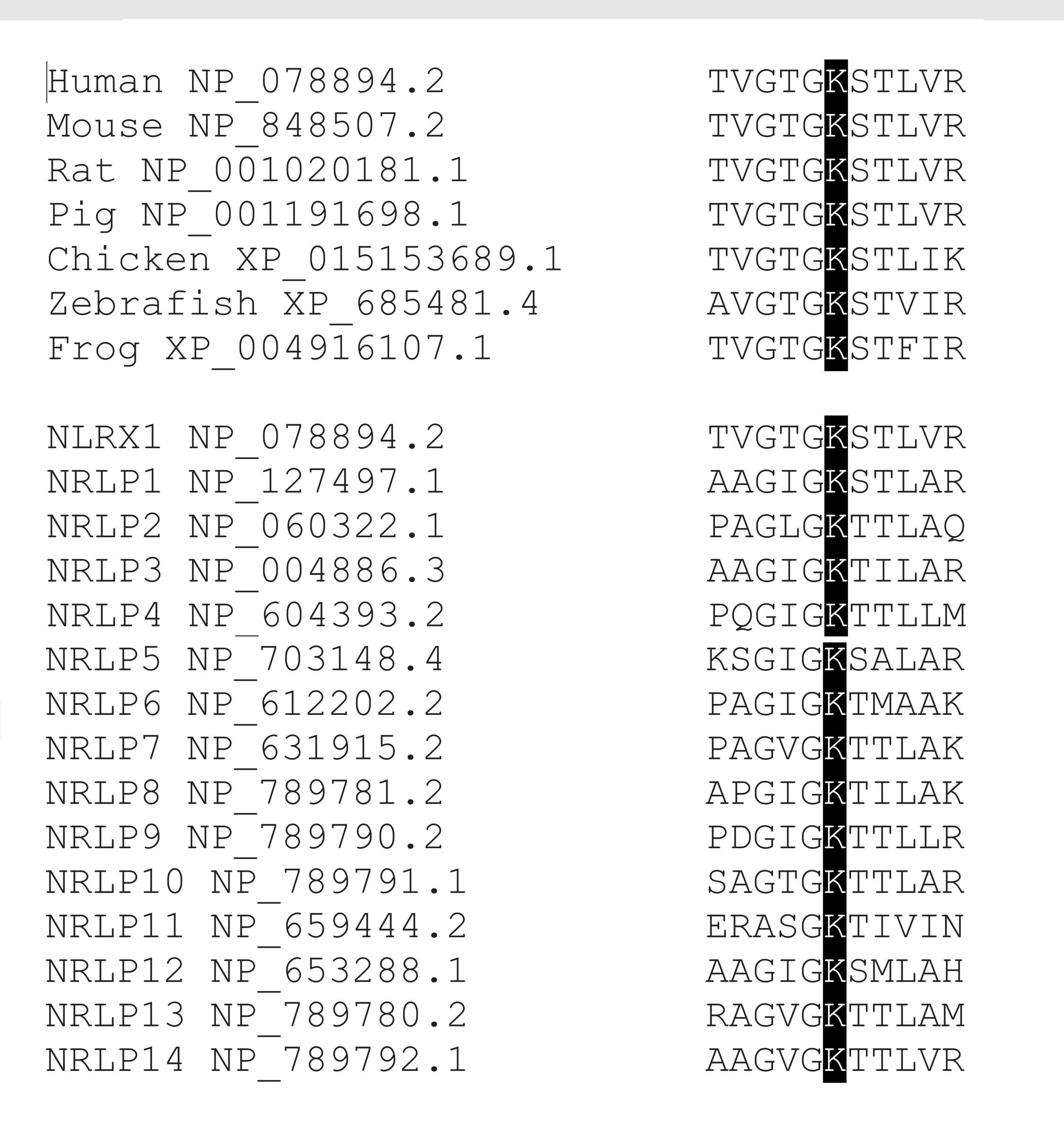

Supplement: S9 Fig — Organism and RefSeq accession numbers are provided for orthologs and gene name and RefSeq accession numbers for human paralogs, which were obtained from Ensembl release 91. Evolutionarily conserved positions for the NLRX1 K172N mutation is highlighted in black. (TIF) [file pbio.3000451.s013.tif]

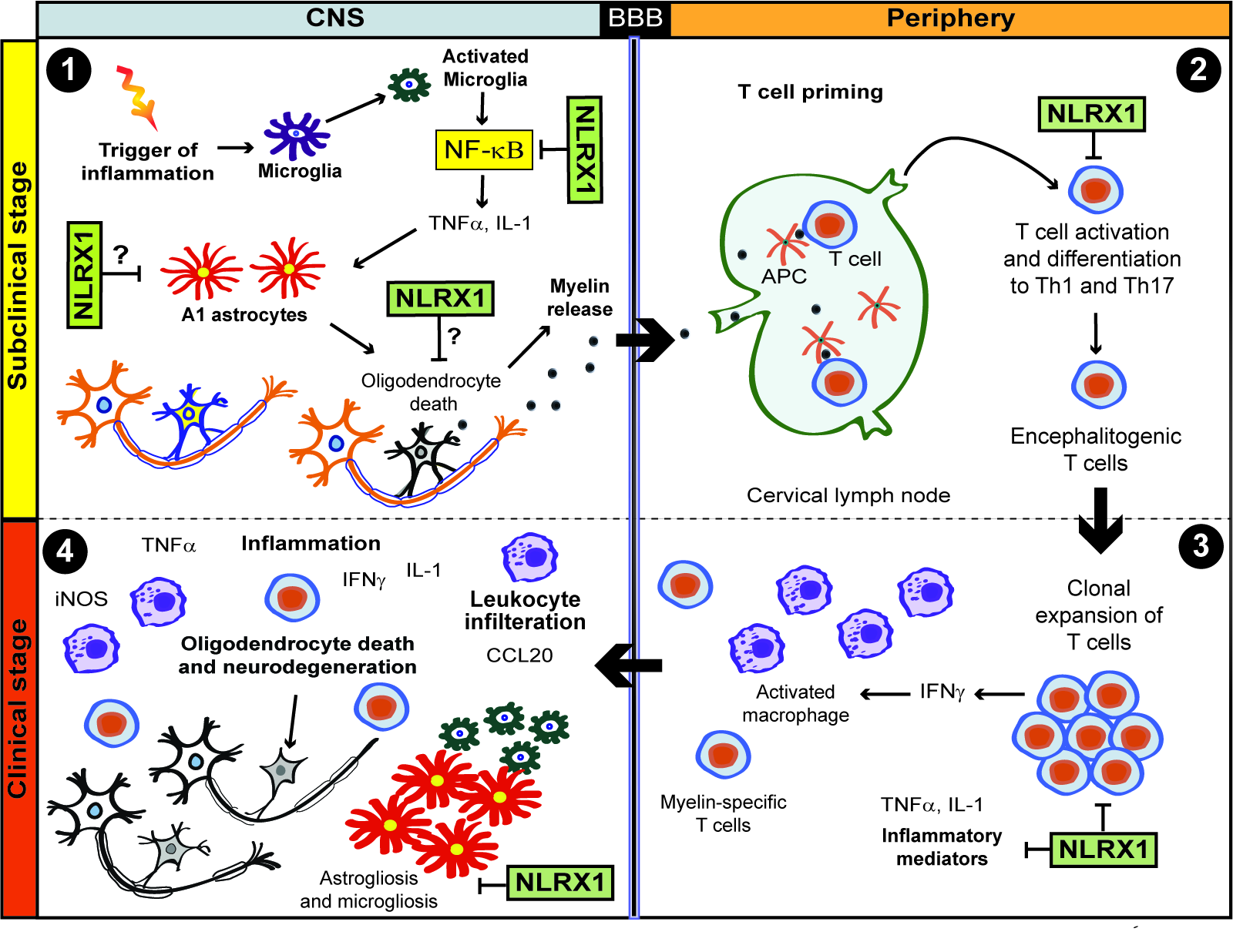

Supplement: S10 Fig — Failure to maintain proper inflammatory balance in Nlrx1−/− microglia produces a milieu that promotes toxic A1 astrocyte phenotype (A1). This results in damage to neurons and oligodendrocytes and creates a T-cell chemoattractant gradient. Upon activation and differentiation in secondary lymphoid organs, Th1-biased T cells migrate to the CNS and induce autoimmune attack. Subclinical stage, phase 1: from previous studies, we know that unknown factors trigger the inflammatory pathways in microglia [60]. This may lead to the production of inflammatory cytokines such as TNFα and IL-1β that promote generation of neurotoxic A1 astrocytes. As a result, a limited number of oligodendrocytes die, and myelin antigen is drained to the deep cervical lymph nodes. At the same time, A1 astrocytes increase expression of T-cell chemokines such as CCL20. Subclinical stage, phase 2: in the lymph nodes, autoreactive T cells proliferate and differentiate into encephalitogenic T-cell subsets (Th1, Th17). Clinical stage, phase 3: clonal expansion of autoreactive encephalitogenic T cells, activation of myeloid cells such as monocytes and macrophages, and production of inflammatory mediators leads to their excavation into the CNS. Clinical stage, phase 4: activated T cells and monocyte/macrophages infiltrate into the CNS, interact with hyperactivated glial cells and boost the inflammation, resulting in reactive gliosis, progressive inflammatory demyelination, neurodegeneration, and eventually the appearance of neurological symptoms. NLRX1 has a broad range of regulatory activity, preventing the onset of clinical signs at the levels of the CNS and periphery. Whether Nlrx1−/− astrocytes themselves are prone to differentiation to the neurotoxic phenotype or Nlrx1−/− oligodendrocytes are intrinsically susceptible to cell death is still unknown. The sequence of events from a preclinical to a clinical stage warrants further investigation. CCL20, C-C motif chemokine ligand 20; CNS, central nervous s [file pbio.3000451.s014.tif]
